# Weighted gene co-expression network analysis identifies key hub genes and pathways in acute myeloid leukemia

**DOI:** 10.3389/fgene.2023.1009462

**Published:** 2023-02-27

**Authors:** Xinfeng Wang, Akhilesh K. Bajpai, Qingqing Gu, David G. Ashbrook, Athena Starlard-Davenport, Lu Lu

**Affiliations:** ^1^ Department of Hematology, Affiliated Hospital of Nantong University, Nantong, Jiangsu, China; ^2^ Department of Genetics, Genomics, and Informatics, University of Tennessee Health Science Center, Memphis, TN, United States

**Keywords:** WGCNA, leukemia, AML, systems genetics, protein interaction, gene expression, co-expression

## Abstract

**Introduction:** Acute myeloid leukemia (AML) is the most common type of leukemia in adults. However, there is a gap in understanding the molecular basis of the disease, partly because key genes associated with AML have not been extensively explored. In the current study, we aimed to identify genes that have strong association with AML based on a cross-species integrative approach.

**Methods:** We used Weighted Gene Co-Expression Network Analysis (WGCNA) to identify co-expressed gene modules significantly correlated with human AML, and further selected the genes exhibiting a significant difference in expression between AML and healthy mouse. Protein-protein interactions, transcription factors, gene function, genetic regulation, and coding sequence variants were integrated to identify key hub genes in AML.

**Results:** The cross-species approach identified a total of 412 genes associated with both human and mouse AML. Enrichment analysis confirmed an association of these genes with hematopoietic and immune-related functions, phenotypes, processes, and pathways. Further, the integrated analysis approach identified a set of important module genes including *Nfe2*, *Trim27*, *Mef2c*, *Ets1*, *Tal1*, *Foxo1*, and *Gata1* in AML. Six of these genes (except *ETS1*) showed significant differential expression between human AML and healthy samples in an independent microarray dataset. All of these genes are known to be involved in immune/hematopoietic functions, and in transcriptional regulation. In addition, *Nfe2*, *Trim27*, *Mef2c*, and *Ets1* harbor coding sequence variants, whereas *Nfe2* and *Trim27* are *cis*-regulated, making them attractive candidates for validation. Furthermore, subtype-specific analysis of the hub genes in human AML indicated high expression of *NFE2* across all the subtypes (M0 through M7) and enriched expression of *ETS1*, *LEF1*, *GATA1*, and *TAL1* in M6 and M7 subtypes. A significant correlation between methylation status and expression level was observed for most of these genes in AML patients.

**Conclusion:** Findings from the current study highlight the importance of our cross-species approach in the identification of multiple key candidate genes in AML, which can be further studied to explore their detailed role in leukemia/AML.

## 1 Introduction

Leukemia is characterized by the uncontrolled proliferation of white blood cells, initially occurring in bone marrow, and subsequently expanding through the blood (Chennamadhavuni et al., 2022). With a global incidence rate of 2.5% (474,519 cases) and death rate of 3.1% (311,594 cases), leukemia was the 15th most commonly diagnosed and 11th leading cause of cancer-related mortalities worldwide in 2020 (Sung et al., 2021). Furthermore, accounting for 28% of all cancer-related cases, it is estimated to be the most common childhood cancer in the United States in 2022 (Siegel et al., 2022). Depending on the rate of spreading, leukemia can either be classified as acute or chronic, whereas, the myeloid or lymphoid classification signifies the lineage of the transformed cells (Chennamadhavuni et al., 2022). There are four predominant subtypes of leukemia: acute lymphocytic leukemia (ALL), acute myelogenous leukemia (AML), chronic myelogenous leukemia (CML), and chronic lymphocytic leukemia (CLL) (Chennamadhavuni et al., 2022). While ALL is the most common leukemia in pediatrics, accounting for up to 80% of cases, AML is most common in adults. CLL occurs due to the proliferation of monoclonal lymphoid cells and is most common in people aged 60–70 years (Chennamadhavuni et al., 2022). The various risk factors for leukemia include age, genetic predisposition, infections, and environmental exposures (Stieglitz and Loh, 2013; Thakkar et al., 2014; Bispo et al., 2020). Germline mutations and chromosomal abnormalities, including rearrangements, translocations, and deletions are known to be the most common causes of both acute and chronic forms; however, the exact cause of most leukemia subtypes is unknown. Although the last few decades have witnessed great progress in studying the malignant transformation of hematopoietic cells, there is still a gap in understanding the underlying mechanisms and molecular factors.

Large-scale techniques, such as microarray and RNA sequencing, have made it possible to study the genome-wide expression of genes associated with a particular physiological or pathological condition. Furthermore, weighted gene co-expression network analysis (WGCNA) and similar network-based approaches are increasingly being used for exploring the correlation patterns among genes based on their expression. WGCNA can be used for finding clusters (modules) of highly correlated genes, and hence, for identifying candidate biomarkers or therapeutic targets (Langfelder and Horvath, 2008). This approach has been successfully used to identify hub genes and gene modules in various experimental (Maertens et al., 2018) and disease conditions (Liu et al., 2019). Additionally, a few reports have employed WGCNA to identify prognostic factors in leukemia (Chen et al., 2019; Ye et al., 2019). Chen et al. (2019) used WGCNA, and identified the long non-coding RNA, *LOC646762* as a potential biomarker for predicting the survival of adult AML patients, as well as for risk stratification. Similarly, Ye et al. (2019) identified cysteine-rich intestinal protein 1 (*CRIP1*) as a potential prognostic biomarker in AML patients using the WGCNA co-expression network analysis.

In the current study, we combined the correlation network analysis approach with systems genetics to identify key hub genes in leukemia. The systems genetics data was generated from the BXD recombinant inbred (RI) mice, derived from crosses between C57BL/6J (B6) and DBA/2J (D2) inbred strains (Ashbrook et al., 2021). The BXD family was started 50 years ago and is now comprised of ∼150 fully sequenced inbred strains. This family segregates for ∼6 million sequence variants scattered across the genome; exceeding the number of variants segregating in many human populations. The initial set of 32 BXD RI strains was used to map Mendelian traits (Taylor et al., 1973; Ashbrook et al., 2021), however, currently these strains that have been expanded to 152 lines by our group in the last 20 years (Peirce et al., 2004; Ashbrook et al., 2021), are used for mapping complex traits, such as behavior (Ashbrook et al., 2018), immune-function (Xu et al., 2020a), brain structure (Rosen et al., 2009), and various diseases, including cancer (Zhu et al., 2020; Wang et al., 2022), metabolic and cardiovascular diseases (Koutnikova et al., 2009; Xu et al., 2020b). Furthermore, the availability of the large-scale omics data (i.e., transcriptomic, proteomic, and metabolomic) from multiple tissues of BXD mice associated with different experimental and physiological conditions provide a powerful genetic resource to decode the molecular mechanisms and genetic regulatory networks. Bystrykh et al. (2005) combined large-scale mRNA expression analysis and gene mapping to identify genes and loci that control hematopoietic stem cell (HSC) function. A study by Henckaerts et al. (2004) used BXD strains to identify genetic regulators associated with the proliferative capacity of HSCs and progenitor cells. Cahan and Graubert (2010) integrated genomics strategies in inbred mice strains to identify novel factors that might contribute to therapy-related acute myeloid leukemia (t-AML) susceptibility. Recently, we also (Wang et al., 2022) revealed the role of *Bcl2* in leukemia pathogenesis through systems genetics by taking advantage of the data generated in BXD RI strains.

Here, we used WGCNA to identify co-expression modules that were significantly correlated with AML. The association of the module genes with leukemia was further confirmed based on their differential expression pattern in a mouse model of AML. Using protein-protein interaction network analysis, we analyzed the significantly correlated modules, and identified genes that may be important in leukemia pathogenesis. By incorporating a systems genetics approach, the module genes were further narrowed down to identify key hub genes in AML. Our approach not only combines human and mouse expression data but also integrates network analysis and systems genetics to shortlist the candidate genes that may have key roles in leukemia pathogenesis.

## 2 Materials and methods

### 2.1 Microarray data analysis

The microarray raw data files (*.CEL* files) for AML and healthy controls corresponding to *Homo sapiens* (accession: GSE9476 and GSE14924) and *Mus musculus* (accession: GSE13690) were downloaded from the GEO database (Barrett et al., 2013). The dataset GSE9476, corresponding to human was used for WGCNA analysis. It contained a total of 64 samples (38 healthy and 26 AML) that were profiled on an Affymetrix Human Genome U133A array. The mouse dataset containing 34 AML samples from five cohorts of mice where leukemia was initiated using distinct MLL fusion oncogenes and four normal bone marrow samples (profiled on Affymetrix Mouse Genome 430 2.0 Array) was used for differential expression analysis. The raw data were background corrected and normalized using the *RMA* method (Irizarry et al., 2003) in R, which resulted in *log2*-transformed signal intensities. Differential expression of the probe-sets was performed using the *limma* package (Ritchie et al., 2015). The probes that did not match to any gene symbol were excluded. Genes with a fold change ≥1.5 and Benjamini-Hochberg adjusted *p* < 0.05 were considered statistically significant. An independent human microarray dataset, GSE14924 (containing 20 AML and 21 healthy samples) was used for validation.

### 2.2 Weighted gene co-expression network analysis (WGCNA)

WGCNA is a method that is used to construct the co-expression network of genes and to explore the association between phenotypes and gene expression levels. We used WGCNA package (Langfelder and Horvath, 2008) in R to construct a co-expression network and identify significant modules using the human AML gene expression dataset, GSE9476. All the samples were used for network analysis based on the hierarchical clustering result (Supplementary Figure S1). Briefly, the weighted adjacency matrix was constructed using the soft-thresholding power (*β*) of 12 to attain scale-free topology. The adjacency matrix was converted to topologically overlapping matrix and then to the dissimilarity matrix. The dissimilarity matrix was used to hierarchically cluster the genes. The clustered genes were then assigned to different modules. The modules were identified by dynamic tree cutting and with a minimum module size of 30. Further, to quantify co-expression similarity of entire modules, we calculated their eigengenes and clustered them on their correlation. A correlation of 75% (distance threshold of 0.25) was used to merge similar modules. The module eigengenes were then correlated with AML, and association with a *p* < 0.05 were considered statistically significant. The genes corresponding to significantly correlated modules and with a significant differential expression between AML and healthy mouse model were considered for further analysis.

### 2.3 Functional enrichment analysis

The genes corresponding to significantly correlated modules and with a significant differential expression between AML and healthy mouse model were considered for functional enrichment analysis including Gene Ontology (GO), Kyoto Encyclopedia of Genes and Genomes (KEGG) pathway and Mammalian Phenotype Ontology (MPO) enrichment analyses. The GO and KEGG pathway enrichment analyses were performed using *clusterProfiler* R package (Yu et al., 2012) with default parameters, whereas MPO analysis was performed using WebGestalt (Liao et al., 2019) with the reference background as “protein coding genes” and a threshold of minimum five genes/transcripts per category. Annotations with a *p* < 0.05 were considered statistically significant. Benjamini-Hochberg correction was used for controlling the false discovery rate (FDR). Additionally, the Mouse Genome Informatics (MGI) database (Law and Shaw, 2018) (http://www.informatics.jax.org/) was used to retrieve hematopoietic or immune phenotype related information for the selected genes.

### 2.4 Protein-protein interaction (PPI) analysis

The significant module genes that were also differentially expressed between AML and healthy mice were considered for PPI analysis using the NetworkAnalyst tool (Zhou et al., 2019). The International Molecular Exchange (IMEx) interactome database (Breuer et al., 2013) within NetworkAnalyst was used for obtaining the interactions of queried (or seed) proteins. The “first-order” PPI network, which includes interactions among the seed proteins as well as their direct interactors was considered.

### 2.5 Transcription factor (TF) analysis

The complete list of TFs was downloaded from the TRRUST database (https://www.grnpedia.org/trrust/) and was matched with the key module genes to identify possible transcriptional regulators. TRRUST is a manually curated database containing human and mouse transcriptional regulatory networks (Han et al., 2018). Additionally, the TF enrichment analysis was performed using the Enrichr web tool (https://maayanlab.cloud/Enrichr/) (Chen et al., 2013), where we used “ChEA 2016” and “TRRUST 2019” datasets to obtain the significantly enriched TFs with a Fisher’s exact test *p* < 0.05. ChEA contains TF-target data from ChIP-seq based studies (Lachmann et al., 2010). From both these databases, TFs corresponding to only mouse were considered for further analysis.

### 2.6 Clinicopathological validation and methylation analysis

We used UALCAN (Chandrashekar et al., 2022) to associate the expression of the selected hub genes with various clinicopathological characteristics, such as overall survival, patient gender, subtype of the disease, and mutation status of *FLT3* gene in AML patients. This database uses gene expression and clinical data from The Cancer Genome Atlas (TCGA) project (Cancer Genome Atlas Research Network et al., 2013). Furthermore, the correlation between methylation and expression profile of the candidate genes was performed using Shiny Methylation Analysis Resource Tool (SMART) platform (Li et al., 2019). SMART (http://www.bioinfo-zs.com/smartapp/) is a user-friendly web application for comprehensively analyzing the human DNA methylation data from the TCGA database. The “Correlation” feature performs pair-wise correlation analysis to explore the relationships between the gene expression and DNA methylation using *Pearson* method. A total of 170 AML samples with gene expression and methylation data were included for correlation analysis. The SMART tool uses the log2-scaled (TPM + 1) value (gene expression) and Beta-value (methylation) for correlation calculation.

### 2.7 Expression QTL (eQTL) mapping

eQTL mapping was conducted with WebQTL in GeneNetwork. The 7,320 informative SNP markers which segregated in BXD RI strains were used for interval mapping. Likelihood ratio statistics (LRS) were used to assess the association between differences in gene expression and differences in particular genotype markers. Genome-wide significance (*p-value* < 0.05) was calculated based on 2000 permutation tests. eQTL mapping in this study was used to determine whether differently expressed genes between AML and healthy control are *cis*-regulated. *Cis*-regulated genes are located within 5–10 Mb interval of the peak SNP location and are therefore more likely to be controlled by these variants and have downstream effects on the expression of other genes and phenotypes. The transcriptomic data corresponding to myeloid cells of BXD RI mice used in the current study was generated by our collaborators earlier and can be accessed through our GeneNetwork website (http://www.genenetwork.org/) with the identifier “UMCG Myeloid Cells ILM6v1.1 (Apr09) transformed” under the group name “BXD Family” and type “Hematopoietic Cells mRNA”. More details on the dataset can be found here: http://gn1.genenetwork.org/webqtl/main.py?FormID=sharinginfo&GN_AccessionId=144.

### 2.8 Whole genome sequencing (WGS) of DBA/2J mouse

DBA/2J (D2) mouse is one of the parental strains of BXD RI strains. The WGS of D2 was carried out by our group earlier as described in previous publication (Sasani et al., 2022). Briefly, D2 mouse were euthanized using isoflurane. Spleen tissue was collected and placed in a −80°C freezer for subsequent analysis. All DNA extraction, library preparations and sequencing were carried out by HudsonAlpha (Huntsville, AL, United States). The FASTQ files were aligned to the mm10/GRCm38 C57BL/6J (B6) reference genome using the 10X LongRanger software (v2.1.6). Variant calling was carried out on aligned BAM files using GATK version v3.8-1-0. The final variants were identified by three distinct call sets: 1) variants identified in DBA/2J in this study, 2) variants identified in DBA/2J in our previous study (Wang et al., 2016), and 3) variants identified in DBA/2J in the Sanger Mouse Genomes Project. This generated a set of variants including 3,972,727 SNPs, 404,349 deletions and 365,435 insertions between the D2 and B6 reference sequences.

## 3 Results

### 3.1 Identification of co-expression modules using human AML gene expression data

**FIGURE 1:**
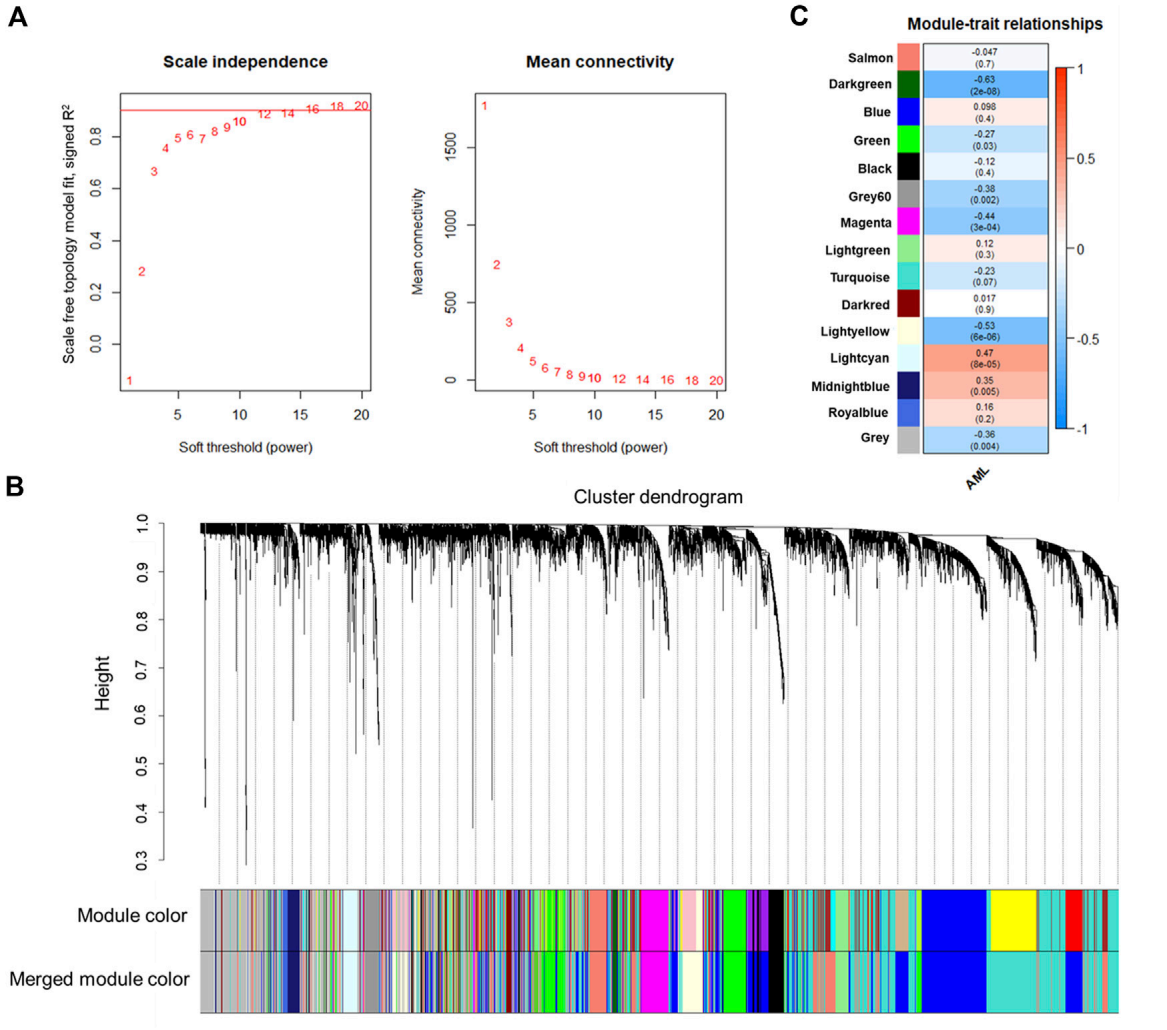
WGCNA analysis of the human AML expression data. **(A)** Soft-thresholding index *R* ^2^ or mean connectivity (*y-axis*) as a function of different *β* thresholds (*x-axis*). A *β* of 12 was selected for constructing the network (indicated by a red line in the plot). **(B)** Gene dendrogram obtained by clustering the dissimilarity based on consensus Topological Overlap. The color rows show the preliminary (module color) and the merged module assignments (merged module color). A total of 23 preliminary modules were detected by clustering 6,200 genes. Merging the modules with a distance threshold of 0.25 resulted in 15 modules. **(C)** Correlation between module eigengenes and the clinical trait of the samples (presence or absence of the disease). Associations with a *p* < 0.05 were considered significant. The numbers in the heatmap represent the correlations of the corresponding module eigengenes and clinical trait, with *p*-values shown in parentheses. The color intensity indicates the correlation.

The co-expression network construction was performed using the human AML gene expression data with WGCNA R package by using a soft thresholding power of 12 (Figure 1A). We used the top 50% (6,200) genes with highest variance for the co-expression network analysis. As shown in Figure 1B, the 6,200 genes were clustered into a total of 15 co-expression modules, each of which contained a different number of genes: turquoise (*n* = 1,506), blue (*n* = 1,210), grey (*n* = 906), salmon (*n* = 787), green (*n* = 461), lightyellow (*n* = 296), black (*n* = 235), magenta (*n* = 215), midnightblue (*n* = 113), lightcyan (*n* = 112), grey60 (*n* = 110), lightgreen (*n* = 105), royalblue (*n* = 64), darkred (*n* = 43), and darkgreen (*n* = 37). The module represented by turquoise color had the most genes, whereas that represented by darkgreen color contained the fewest genes. The grey module contained the unassigned genes; hence it was excluded from further analysis.

### 3.2 Modules significantly correlated with human AML and their expression in mouse model

**FIGURE 2:**
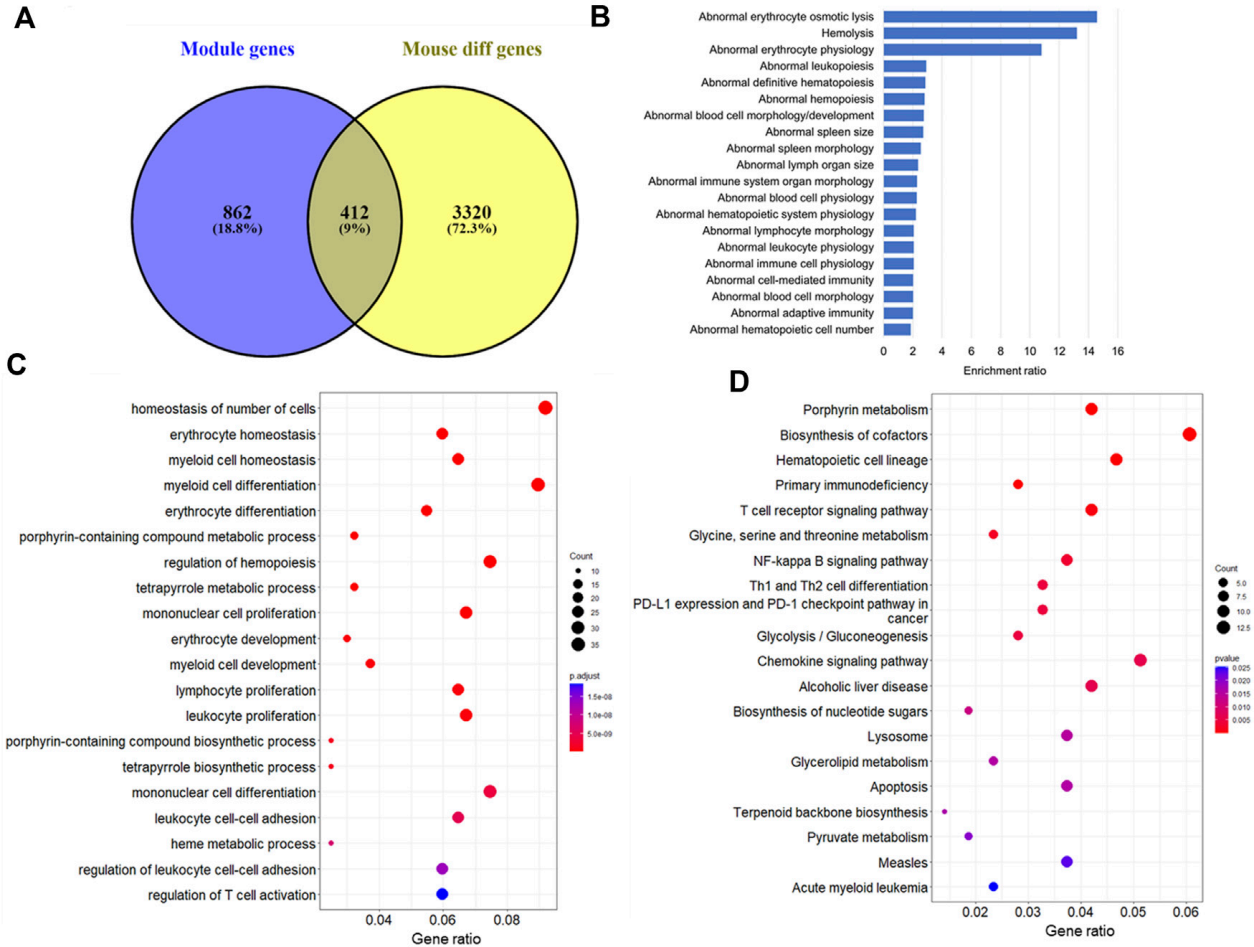
Functional analysis of the module genes that were differentially expressed between AML and healthy mice. **(A)** Number of mice orthologs that were part of significant co-expression modules as well as differentially expressed between AML and healthy mouse (a total of 412 genes were found common between both the lists). Top 20 **(B)** Mammalian Phenotype Ontologies, **(C)** Gene Ontology biological processes, and **(D)** KEGG pathways significantly enriched by the 412 genes.

**FIGURE 3:**
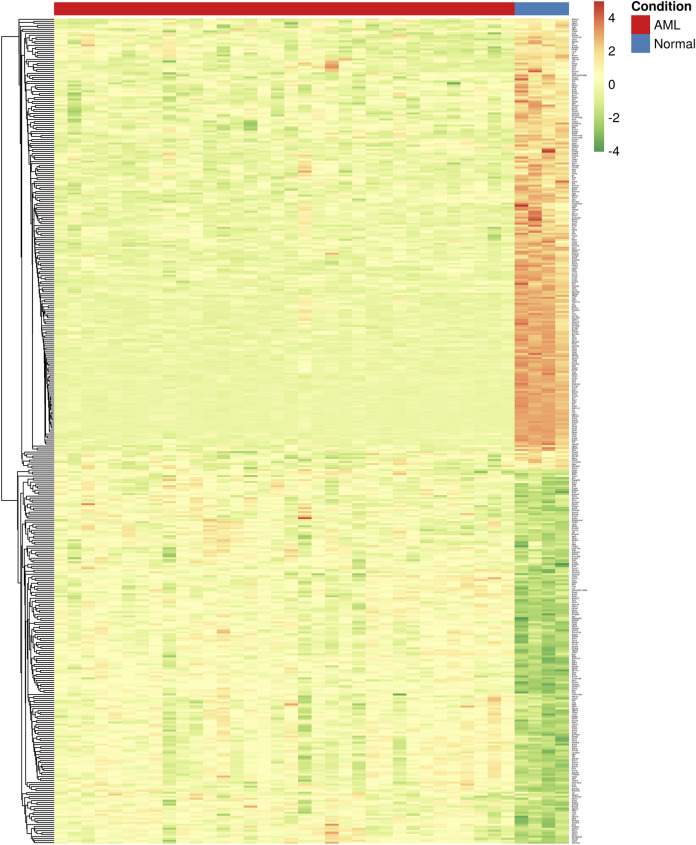
Heatmap showing the differential expression of key module genes (*n* = 412) in mouse. Each column is a sample, and each row is a gene. The color intensity indicates the level of expression of a specific gene in a particular sample (red indicates high expression and green indicates low expression).

Furthermore, module-trait analysis identified seven co-expression modules that were significantly correlated with human AML phenotype (Figure 1C). These included green (*r* = −0.27, *p* = 0.034), lightyellow (*r* = −0.53, *p* = 6.23E-06), magenta (*r* = −0.44, *p* = 0.00026), midnightblue (*r* = 0.35, *p* = 0.0046), lightcyan (*r* = 0.47, *p* = 7.58E-05), grey60 (*r* = −0.38, *p* = 0.0018), and darkgreen (*r* = −0.63, *p* = 2.19E-08) modules, and contained a total of 1,344 human genes, which mapped to 1,274 mouse orthologs. Among the 1,274 mouse ortholog genes that were part of the significant co-expression modules, 412 were also found to be significantly differentially expressed between AML and healthy mice (Figure 2A, Figure 3). Thus, these genes may play an important role in the modulation of various processes and pathways related to AML in both human and mouse. Hence, we considered these 412 genes for further analysis. Among these, a total of 187 genes were upregulated (*log2FC*: 0.58–4; *adj. p-value* < 0.05) and 225 genes were found to be downregulated in AML compared to healthy mouse (*log2FC*: 0.58–8.9; *adj. p-value* < 0.05). Interestingly, all the significant co-expression modules contributed to the list of differentially expressed genes in mouse, and as expected, most of the differential genes were from the “green” module (*n* = 113), followed by “magenta” module (*n* = 88). Furthermore, 80, 55, 44, 24, and 8 genes were found to be differentially expressed in mouse from “lightyellow,” “grey60,” “lightcyan,” “darkgreen,” and “midnightblue” modules, respectively. Supplementary File S1 lists the significant module genes in human and their differential expression in mouse.

### 3.3 Key module genes are involved in functions and pathways related to blood and immune system physiology

The 412 genes that were found to be important in both human and mouse AML were further explored to understand their involvement in leukemia pathology through functional and pathway analyses. The functional analysis indicated enrichment of various pathways and processes associated with blood physiology. A total of 166 MPOs were found to be significantly represented by the key module genes with an *adj. p* < 0.05. It is noteworthy that most of these MPOs (approximately >90%) were associated with blood cell and hematopoietic system-related physiology or were involved in immune system related functions (Figure 2B). “Abnormal blood cell morphology/development” was the most significant MPO (*adj. p* = 2.73E-11) containing a total of 68 genes. The other important MPOs included “abnormal hematopoietic system physiology” (*n* = 88; *adj. p* = 1.49E-10), “abnormal immune system organ morphology” (*n* = 72; *adj. p* = 5.01E-09), “abnormal spleen morphology” (*n* = 59; *adj. p* = 7.04E-09), “abnormal adaptive immunity” (*n* = 73; *adj. p* = 7.21E-07). Additionally, a few of the MPOs were related to apoptosis, abnormal kidney iron level, and urinary system related functions. A complete list of significant MPOs is provided in Supplementary File S2.

Similarly, GO analysis indicated the enrichment of several biological processes (BP) related to immune system and blood cell physiology. Overall, more than 700 GO-BPs were found to be significantly enriched by the key module genes with an *adj. p* < 0.05. The processes, such as “homeostasis of number of cells” (*n* = 37; *adj. p* = 1.35E-17), “myeloid cell differentiation” (*n* = 36; *adj. p* = 5.65E-15), “erythrocyte development” (*n* = 12; *adj. p* = 3.40E-10), and “leukocyte proliferation” (*n* = 27; *adj. p* = 4.20E-10) were among the highly significant GO annotations (Figure 2C). Pathway analysis revealed the significant enrichment of five KEGG pathways with an adjusted *p* < 0.05. These pathways include “porphyrin metabolism” (*n* = 9; *adj. p* = 0.0002), “biosynthesis of cofactors” (*n* = 13; *adj. p* = 0.007), “hematopoietic cell lineage” (*n* = 10; *adj. p* = 0.007), “primary immunodeficiency” (*n* = 6; *adj. p* = 0.013), and “T-cell receptor signalling pathway” (*n* = 9; *adj. p* = 0.042). Other important pathways that were affected by the key module genes were signalling pathways, such as NF-kappa B, chemokine, and FoxO signaling, and acute myeloid and chronic myeloid leukemia pathways. Furthermore, it is noteworthy that the key module genes significantly represented multiple metabolic pathways. A few of the top pathways include “Glycolysis/Gluconeogenesis,” “Glycine, serine and threonine metabolism,” and “Biosynthesis of cofactors” (Figure 2D). A complete list of significant GO annotations and KEGG pathways is provided in Supplementary File S2.

### 3.4 TF analysis reveals factors regulating key module genes in AML

The TF analysis was performed to a) determine whether there are any regulatory factors among the key module genes, and b) identify the key regulators that may be involved in the regulation of these module genes. Our analysis revealed that among the 412 module genes that were identified based on both human and mouse expression data, ∼9% (36 proteins) were found to act as transcription factors, based on the manually curated TF-target relationship from the TRRUST database. Furthermore, to reveal whether any of these TFs control the expression of the 412 module genes, we performed TF-enrichment analysis using the ChEA and TRRUST datasets in Enrichr. Our results showed significant enrichment of 80 TFs (union list of ChEA and TRRUST enrichment) that may be involved in the regulation of the 412 genes (Supplementary File S3). Interestingly, 12 TFs (MYC, KLF1, NFE2, GFI1B, FOXO3, TAL1, GATA1, CREM, FOXO1, NFE2L2, TCF7, and ETS1) were found to be among the 36 TFs within the key module genes. Furthermore, four TFs (GFI1B, TAL1, GATA1, and FOXO1) were found to be enriched by both ChEA and TRRUST datasets. Thus, these TFs may be important in regulating the expression of the genes associated with immune function and blood physiology in both human and mouse.

### 3.5 Identification of important nodes through PPI analysis of the key module genes

**FIGURE 4:**
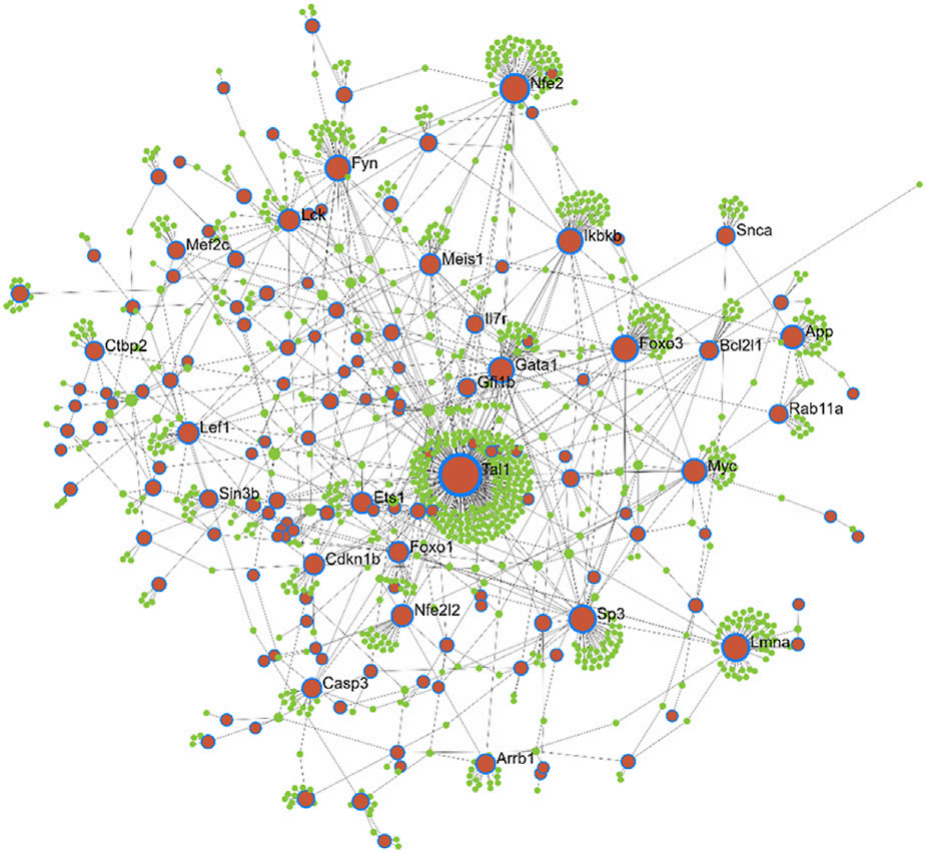
Protein-protein interaction network of the key module genes differentially expressed between AML and healthy mouse. Brown nodes with blue borders: module genes (also known as seed proteins). Green nodes: proteins directly interacting with the seed proteins. Node size indicates increasing degree.

Protein-interaction analysis of the key module genes was performed to identify important nodes (proteins) within the 412 module genes. While constructing the PPI network, interactions between the seed proteins as well as their first interactors were considered. Thus, the network analysis resulted in a total of 11 subnetworks (containing at least three nodes), among which, the first subnetwork had highest number of nodes and edges, and hence, it was considered for further analysis (Figure 4). The selected subnetwork (henceforth referred to as “PPI network”) included 1,095 nodes that were connected with 1,329 edges (interactions). Of the 1,095 nodes, 150 were seed proteins, and the remaining were their direct interactors. The average node degree was 2.4, whereas the average betweenness score of the nodes was found to be ∼1943. The analysis of the PPI network indicated TAL1 to be the most important node having 251 interacting partners (degree) and having the highest betweenness score. The other important nodes in the network were NFE2, LMNA, SP3, FOXO3, GATA1, IKBKB, FYN, MYC, APP, and NFE2L2, each having a node degree of at least 25. There were a total of 37 proteins that had a node degree of at least 10, and interestingly, 33 of these were seed proteins.

### 3.6 Identification of leukemia hub genes

**FIGURE 5:**
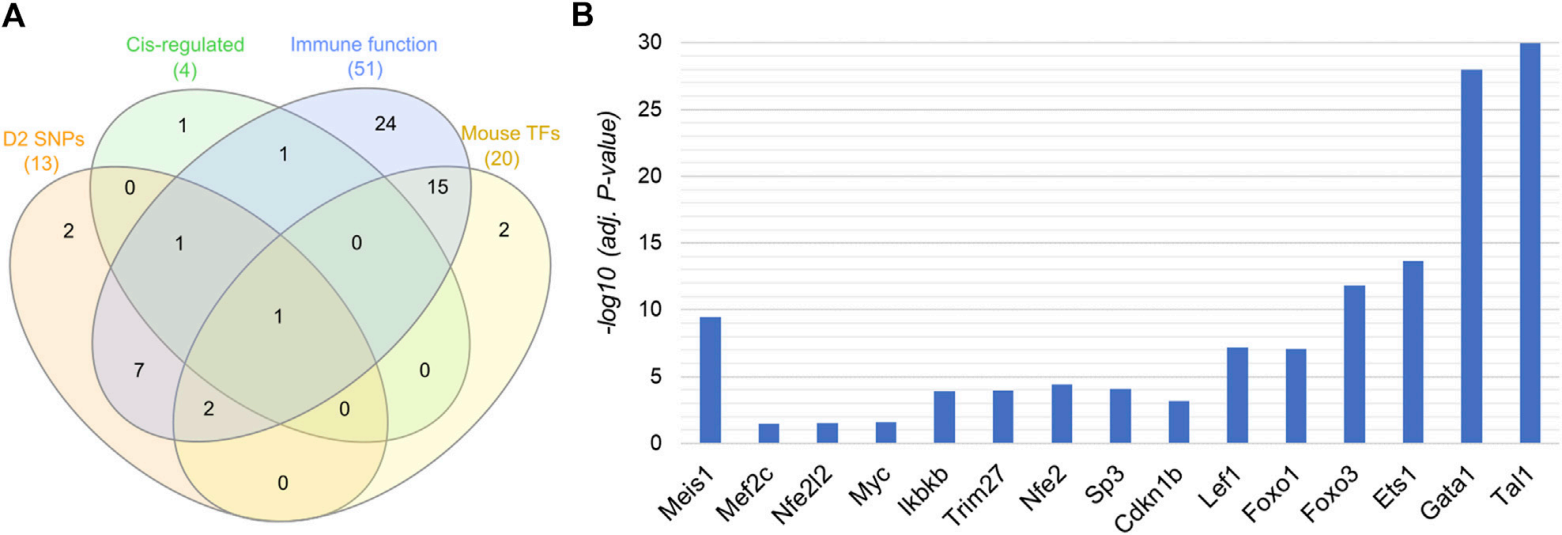
Genes shared across different functional categories. **(A)** Genes with a node degree of at least 5 (*n* = 61) are shown in the Venn diagram. Genes were selected based on the following categories: harbouring coding SNPs, *cis*-regulated in BXD strains, acting as TFs, and associated with immune/hematopoietic system related function or phenotype. **(B)** Statistical significance of selected genes involved in immune functions along with at least one of the other three functional categories (shown in the Venn diagram). −log10 (adj. *p*-value) in *y*-axis represents the significant differential expression of the genes between mouse AML and normal control. A −log10 (adj. *p*-value) of 1.3 is equal to an actual adjusted *p*-value of 0.05.

The leukemia hub genes in the current study were identified based on multiple criteria. We integrated different data types including PPI, mutation, *cis*-regulation, and functional data to identify important hub genes in leukemia. First, we focused our analysis on the seed proteins that were part of the PPI network (*n* = 150). Among the seed proteins, a total of 61 genes were connected to at least five other proteins (node degree ≥5), 33 genes had coding mutations in D2 parental mice strain, 10 genes were *cis*-regulated, and 107 genes were involved in the hematopoietic system/immune system phenotypes/diseases or cancer. We considered the genes having a node degree of ≥5 (*n* = 61) for further selection. Among these, 13 genes had coding SNPs (*Mef2c*, *Ctbp2*, *Kifap3*, *Trim27*, *Atrx*, *Ivns1abp*, *Gbp2*, *Rad21*, *Nfe2*, *Cdkn1b*, *Anxa1*, *Ets1*, and *Arrb1*), four genes were *cis*-regulated (*Tia1*, *Trim27*, *Nfe2*, and *Msl1*), 20 genes were mouse TFs, and 51 genes were involved in immune/hemopoietic-related phenotypes/diseases (Figure 5). Table 1 lists the genes that are involved in immune/hematopoietic-related functions and in at least one of the other three categories.

**TABLE 1:** Selected key genes with a node degree ≥5 in protein interaction network.

Gene symbol	Node degree	D2 SNP	Cis-regulated	TF	Immune function
**Meis1**	**22**	**--**	**×**	**✓**	**✓**
**Mef2c**	**12**	**Splice site**	**×**	**✓**	**✓**
Tia1	5	--	✓	×	✓
Atrx	6	Non-synonymous	×	×	✓
Ivns1abp	6	Non-synonymous	×	×	✓
Satb2	5	--	×	✓	✓
**Nfe2l2**	**25**	**--**	**×**	**✓**	**✓**
**Myc**	**36**	**--**	**×**	**✓**	**✓**
**Ikbkb**	**42**	**--**	**×**	**✓**	**✓**
**Trim27**	**7**	**Non-synonymous**	**✓**	**×**	**✓**
Sin3b	13	--	×	✓	✓
Ddit3	5	--	×	✓	✓
Gbp2	7	Non-synonymous	×	×	✓
Rad21	7	Non-synonymous	×	×	✓
**Nfe2**	**66**	**Non-synonymous**	**✓**	**✓**	**✓**
**Sp3**	**52**	**--**	**×**	**✓**	**✓**
**Cdkn1b**	**21**	**Non-synonymous**	**×**	**×**	**✓**
Xrcc5	8	--	×	✓	✓
**Lef1**	**23**	**--**	**×**	**✓**	**✓**
**Foxo1**	**21**	**--**	**×**	**✓**	**✓**
Anxa1	5	Non-synonymous	×	×	✓
**Foxo3**	**46**	**--**	**×**	**✓**	**✓**
**Ets1**	**22**	**Frame shift**	**×**	**✓**	**✓**
Gfi1b	13	--	×	✓	✓
Arrb1	17	Non-synonymous	×	×	✓
**Gata1**	**42**	**--**	**×**	**✓**	**✓**
**Tal1**	**251**	**--**	**×**	**✓**	**✓**

Note: Important genes based on different categories have been highlighted in bold font. The significance of differential expression of these genes between mouse AML and normal control is shown in Figure 5B, whereas that of human is shown in Supplementary Figure S2.

Our analysis found *Nfe2* to be common to all the four categories, i.e., harbors D2 SNPs, acts as a TF, *cis*-regulated, and is associated with immune-related functions (Figure 5A). It interacted with a total of 66 proteins in the protein interaction network and harbored non-synonymous mutations. Furthermore, *Trim27* and *Mef2c* were common to two other categories in addition to being involved in immune-related functions. *Trim27* contained non-synonymous mutations and was *cis*-regulated, whereas *Mef2c* acts as a TF and had splice-site mutations. *Trim27* interacted with seven proteins in the PPI network, while *Mef2c* had a node degree of 12 (Table 1). *Ets1*, a TF, was another important molecule that not only interacted with 22 proteins, but also harbored frame-shift mutations. A few other genes, such as *Meis1*, *Myc*, *Ikbkb*, *Sp3*, *Cdkn1b*, *Lef1*, *Foxo1*, *Foxo3*, *Gata1*, *Tal1*, *Nfe2l2* interact with multiple proteins (at least 20) in the network, suggesting that they may have important roles in regulating various immune system-related functions and processes. Interestingly, most of these proteins with high node degree act as TFs, which further strengthens their role in immune-system physiology (Table 1). Furthermore, *Myc*, *Ikbkb*, *Lef1* were also found to be involved in the significantly enriched AML pathway. Figure 5B represents the significance of differential expression of a few important genes between AML and control mouse. All the genes were significantly differentially expressed in mouse with an *adjusted p-value* < 0.05. In addition, we validated the differential expression of these genes using an independent human microarray dataset (GSE14924). Except *NFE2L2* and *ETS1*, all the genes showed a significant differential expression between human AML and healthy tissues (Supplementary Figure S2).

### 3.7 Clinicopathological validation and methylation analysis of the hub genes

We sought to validate the important candidate genes that we identified using a combinatorial integrative approach (Figure 5B). The gene expression values were correlated with various clinicopathological characteristics of AML patients using the UALCAN database. The expression values and data related to various clinicopathological characteristics were obtained from the TCGA project. The correlation of the expression of the candidate genes with sex of the patients indicated only *TAL1* to be slightly significantly different between male and female AML patients (Supplementary Figure S3). Based on French-American-British classification system, AML is divided into subtypes M0 through M7, depending upon the type and maturity of leukemia developing cells. Our analysis showed varying expression of the candidate genes across different AML subtypes (Supplementary Figure S4). We observed high expression of *NFE2* across all the subtypes compared to other candidate genes. While the expression of *TRIM27* was consistent across the subtypes, that of *ETS1*, *LEF1*, *GATA1*, and *TAL1* was specifically higher in the M6 (starts in very immature forms of red blood cells) and M7 (starts in immature forms of cells that make platelets) AML subtypes (Supplementary Figure S4). Furthermore, we verified the expression of the candidate genes between AML patients harboring wild-type or mutated FMS-like tyrosine kinase 3 (*FLT3*) sequence. *FLT3* is a type III receptor tyrosine kinase and plays an important role in survival, proliferation, and differentiation of hematopoietic cells, and is most frequently mutated and is a poor prognostic factor in AML patients (Kiyoi et al., 2020). Thus, mutations in this gene may affect the expression and function of downstream targets. Our analysis showed significant difference in the expression of nine candidate genes (*MEIS1*, *MEF2C*, *NFE2*, *LEF1*, *FOXO1*, *FOXO3*, *ETS1*, *GATA1*, and *TAL1*). While *MEIS1* (*p = 0.0055*), *NFE2* (*p = 0.041*), and *GATA1* (*p = 0.0013*) significantly increased, *MEF2C* (*p = 0.0064*), *LEF1* (*p = 2.64E-07*), *FOXO1* (*p = 1.58E-07*), *FOXO3* (*p = 0.0035*), ETS1 (*p = 2.04E-06*), and *TAL1* (*p = 1.8E-04*) significantly decreased in patients with *FLT3*-mutation (Supplementary Figure S5). The survival analysis revealed that high expression of most of the hub genes decreased the overall survival of AML patients, however, the results were not statistically significant (Supplementary Figure S6). Thus, the clinicopathological analysis suggested that the hub genes identified in the current study may have important roles in the development and progression rather than in the detection or prognosis of AML, however it warrants further investigation.

Next, we performed correlation analysis between the methylation levels and expression of the hub genes to understand their regulation in AML patients. Our results revealed that the expression of approximately 50% of the 15 hub genes (*MEIS1*, *NFE2*, *LEF1*, *FOXO1*, *FOXO3*, *ETS1*, and *GATA1*) was significantly correlated with their methylation levels (Table 2, Supplementary Figures S7−S13). Interestingly, all the genes except *ETS1* were found to be negatively correlated. Furthermore, *MEIS1* (*r* = −0.58; *p* < 2.2e-16) followed by *GATA1* (*r* = −0.54; *p* = 1.8e-14) were the most significantly correlated genes. Thus, our results demonstrated that many of the hub genes identified by us may be regulated through methylation of their promoter regions in AML.

**TABLE 2:** Correlation between gene expression and methylation level of candidate genes in AML patients.

Hub gene name	Candidate gene description	Pearson correlation *R*	Correlation coefficient *p*-value
MEIS1	Meis homeobox 1	−0.58	<2.2e-16
MEF2C	myocyte enhancer factor 2C	−0.025	0.75
NFE2L2	nuclear factor, erythroid derived 2, like 2	−0.12	0.13
MYC	myelocytomatosis oncogene	0.012	0.88
IKBKB	inhibitor of kappaB kinase beta	0.11	0.15
TRIM27	tripartite motif-containing 27	0.073	0.35
NFE2	nuclear factor, erythroid derived 2	−0.43	6.1e-09
SP3	trans-acting transcription factor 3	−0.073	0.35
CDKN1B	cyclin-dependent kinase inhibitor 1B	0.02	0.8
LEF1	lymphoid enhancer binding factor 1	−0.19	0.014
FOXO1	forkhead box O1	−0.34	7e-06
FOXO3	forkhead box O3	−0.25	0.00099
ETS1	E26 avian leukemia oncogene 1, 5′ domain	0.35	3.6e-06
GATA1	GATA binding protein 1	−0.54	1.8e-14
TAL1	T cell acute lymphocytic leukemia 1	−0.089	0.25

Note: The correlation analysis between methylation and gene expression was performed using SMART web-application (http://www.bioinfo-zs.com/smartapp/). The “Correlation” feature performs pair-wise correlation analysis to explore the relationship between the expression and DNA methylation, using Pearson method. A total of 170 AML samples with gene expression and methylation data were included for the correlation analysis. SMART uses the log2-scaled (TPM + 1) value (gene expression) and Beta-value (methylation) for correlation calculation. Plots for the significant genes are shown as Supplementary Figures S7–S13.

## 4 Discussion

AML is the most common type of leukemia in adults, with a mean age at diagnosis of 68 (https://seer.cancer.gov/statfacts/html/amyl.html). Despite decades of research, there is still a gap in understanding the molecular mechanisms associated with leukemia and AML in general. In the current study, we used a combined approach of systems genetics and co-expression network analysis to identify key hub genes associated with AML.

WGCNA analysis of human AML expression data resulted in a total of 15 co-expression modules with genes ranging from 37 to 1,500 across different modules. Further, correlation of these modules with AML helped us in narrowing down to seven co-expression modules that were significantly associated with the disease. However, together these seven modules contained a total of 1,344 genes, a large number to perform a focused analysis. Transgenic mouse models of AML have been extensively used to study the molecular mechanisms associated with leukemia (Almosailleakh and Schwaller, 2019). Hence, we sought to use the expression data from an AML mouse model to further shortlist important leukemia-related genes from the co-expression modules. A combined approach of using cross-species data also ensures greater reproducibility of the findings and has been employed in various conditions, including cancer, aging, neurodegenerative disorder, and osteoarthritis (Miller et al., 2010; Mueller et al., 2017; Jin et al., 2019; Podder et al., 2021). While the analysis by Podder et al. (2021) used experimentally verified protein-protein interaction networks and highly enriched conserved biological pathways across four species, the other three studies (Miller et al., 2010; Mueller et al., 2017; Jin et al., 2019) used “modulePreservation” method (Langfelder et al., 2011) in WGCNA to identify the conserved modules across species. This function assesses how well network properties of a module in a reference dataset are preserved in a test dataset based on a number of variables, such as module size, network size and connectivity. The “modulePreservation” function is particularly useful for exploring the conserved pathways or processes across different species (Langfelder et al., 2011). We used differential expression along with WGCNA network analysis to identify hub genes in the current study. Our approach utilized the strength of each of the methods to prioritize the hub genes. While coexpression is an important criterion for selecting the functionally similar genes, differential expression between disease and heathy condition indicates the causal significance of a particular gene. Hence, the genes identified based on such an approach would have higher reliability and confidence of association with AML. Finally, we identified a set of 412 genes that were significantly correlated with human AML based on module-trait relationship as well as exhibited a significant differential expression between AML and healthy mouse model. It should be noted that each of the seven significantly correlated modules contributed to the differentially expressed genes in mouse, further corroborating the module-trait correlation significance, and confirming the importance of the genes in both the species as well as establishing the validity of our approach.

We performed functional and pathway enrichment analysis of the 412 genes to explore their possible roles in leukemia pathology. Our analysis revealed that these genes are associated with various functions and pathways related to hematopoietic and immune system physiology. The annotations, such as “abnormal blood cell morphology/development,” “abnormal hematopoietic system physiology,” and “abnormal immune system organ morphology” were among the top significantly represented MPOs by the module genes. These annotations have been reported to be enriched by the genes modulated in leukemia (Wang et al., 2022), primary human megakaryocytes (Tijssen et al., 2011) or myeloproliferative neoplasms (Contreras Castillo et al., 2021). Furthermore, the GO and KEGG pathway analyses resulted in the enrichment of similar annotations. The GO biological processes, such as “myeloid cell differentiation,” “erythrocyte development,” and “leukocyte proliferation” were significantly enriched by the key module genes, which was in agreement with the results reported in literature in the context of leukemia/AML (Chen et al., 2020; Schieber et al., 2020). Additionally, the pathway analysis revealed enrichment of several leukemia or hematopoiesis related pathways. “Porphyrin metabolism” was found to be the most significantly enriched KEGG pathway and involved 9 of the 412 module genes. Porphyrins, the ring-shaped molecules undergo a series of chemical changes to produce heme, an important component of hemoglobin. Thus, changes in the metabolism of porphyrin by the module genes may affect the production and maturation of erythrocytes. This pathway has been reported to be affected by the deregulated genes in different hematopoietic cancers, including leukemia (Li et al., 2021). The top 20 enriched pathways also included pathways related to immune system, such as hematopoietic cell lineage, and primary immunodeficiency. As expected, “AML pathway” was also in the list of top 20 significant pathways and contained five of the 412 module genes. Additionally, several signaling pathways were found to be associated with the key module genes, most significant ones being “T cell receptor signaling,” “chemokine signaling” and “NF-kappa B signaling” pathways. While the first two are primarily immune response pathways (Gorentla and Zhong, 2012; Sokol and Luster, 2015), NF-kappa B signaling affects the immune system *via* activating a plethora of downstream target genes. In AML, constitutive NF-κB has been observed in 40% of cases and enables leukemia cells to stimulate proliferation and evade apoptosis (Zhou et al., 2015). Because of the large body of evidence supporting its role in malignant transformation, NFκB has been a promising target for various inhibitors in clinical trials for the treatment of AML (Zhou et al., 2015). In the current analysis, eight genes were found to be involved in NFκB signaling, including *Ikbkb*, one of the important molecules in the NFκB signaling pathway (Schmid and Birbach, 2008). Interestingly, *Ikbkb* was also found to be involved in “T cell receptor signaling,” and “chemokine signaling” pathways. Furthermore, multiple biosynthesis/metabolic pathways were represented by the list of key module genes, with seven pathways being in the top 20. Among these, “Biosynthesis of cofactors” was the most significant pathway followed by “Glycine, serine and threonine metabolism.” A significant increase in fructose intake by the cancer cells has been shown to be important for their proliferation and metastasis, in various tissues (Bu et al., 2018; Weng et al., 2018). A recent study by Jeong et al. (2021) explored the mechanisms underlying fructose metabolism in AML cells in glucose-limited conditions and suggested that the *de novo* serine synthesis pathway could be a promising therapeutic target for leukemia. In the current analysis, five module genes (*Alas2*, *Bpgm*, *Alas1*, *Pgam1*, and *Psat1*) were associated with the “Glycine, serine and threonine metabolism” pathway. The “Warburg Effect,” production of energy through a less efficient aerobic glycolysis process (Warburg et al., 1927) is a well-known phenomenon in cancer cells including leukemic blasts, and is correlated with worse prognosis of AML (Herst et al., 2011). Furthermore, a study by Khan et al. (2020) has shown that transport of pyruvate into the mitochondria through MTCH2 has been linked to the survival and differentiation of AML leukemia stem cells (LSCs), and inhibition of MTCH2 results in the differentiation of LSCs and reduced survival of cancer cells (de Beauchamp et al., 2022). Thus, the leukemic cells rewire the metabolic pathways, especially energy metabolism for their growth and survival, supporting the enrichment of various metabolic pathways, especially glucose metabolism by our module genes in the current study.

Our TF analysis provided insights into the regulation of the key module genes correlated with AML. The results revealed GFI1B, TAL1, GATA1, and FOXO1 to be important regulators as they were enriched by two different TF resources. Furthermore, TAL1 interacted with most of the proteins in the protein interaction network. TAL1 is known to be involved in DNA-binding, E-box binding, and histone deacetylase binding. Our differential analysis results indicated it to be significantly downregulated in both mouse and human AML. The role of *Tal1* in leukemia has been reported previously by multiple studies (Li et al., 2012; Thoms et al., 2021). A review by Tan et al. (2019) discusses in detail the regulatory network and downstream target genes controlled by *Tal1* during hematopoiesis and leukemia. *Tal1* is essential for maintaining the multipotency of hematopoietic stem cells (HSCs) and keeping them in quiescence stage. Our functional analysis confirmed the involvement of *Tal1* in various hematopoiesis-related processes and functions, and in homeostasis of a number of immune cells. The other two important TFs were GATA1 and FOXO1. *Gata1* is known to be involved in several processes, including platelet aggregation, regulation of biosynthetic process, and erythrocyte differentiation. Our functional analysis also indicated its involvement in apoptosis. The role of *Gata1* in deregulation of hematopoiesis has been well established (Sportoletti et al., 2019; Goemans et al., 2021), whereas, the role of *Foxo1* in hematopoiesis has only been recently reported (Gurnari et al., 2019; Zheng et al., 2020). Our results revealed the association of *Foxo1* with several signaling pathways, cellular response to oxidative stress, insulin stimulus and carbohydrate metabolism. Thus, *Foxo1* may be involved in AML *via* regulating signaling pathways, such as WNT and NF-kappa B as well as glucose metabolic pathways.

Furthermore, we employed an integrative approach by including systems genetics, protein interaction, and functional information to shortlist the key hub genes in AML. We used BXD mice and their parental strains to perform systems genetics analysis and identify genes associated with molecular mechanisms underlying myeloid cells, and thus, delineate their possible roles in AML pathogenesis. The *cis*-regulated genes exhibit downstream effects on the expression of other genes and phenotypes, acting as important regulators compared to those that are *trans*-regulated. Hence, using myeloid expression data in combination with sequence variants in BXDs, we identified *cis*-eQTLs for the identified AML genes. Similarly, the presence of non-synonymous-variants in the gene sequence between B6 and D2 parents predicts their protein-coding potential in BXD mice. The BXD strains carrying the mutant variant of the gene may have different molecular and/or phenotypic traits than those carrying the wild-type gene. Hence, these strains can be used for exploring the molecular mechanisms associated with the mutant variant of the genes in myeloid cells, and in turn aid in revealing possible pathological mechanisms underlying AML. Although the systems genetics approach does not directly link the genes to AML, it helps in inferring the possible roles of these genes in AML pathogenesis using an isogenic genetic reference population. Furthermore, we validated the hub genes in AML patients by correlating their expression with various clinicopathological characteristics and methylation levels. Our analysis led to the identification of multiple hub genes, including *NFE2*, *TRIM27*, *MEF2C*, and *ETS1* as important candidates. All of these were found to be involved in immune-related functions and harbored deleterious mutations in D2 mice. While *Mef2c* and *Trim27* were significantly upregulated, *Nfe2* and *Ets1* were downregulated in AML versus healthy mice. In addition, *Nfe2* and *Trim27* were found to be *cis*-regulated according to the GeneNetwork database, making them attractive candidates for further study in AML. While the role of NFE2 transcription factor in hematopoiesis has been well-known for a long time (Shivdasani et al., 1995; Catani et al., 2002), its importance in the malignant transformation of blood cells has been relatively ignored. Only recently, a few reports have studied its importance in leukemia (Jutzi et al., 2019; Marcault et al., 2021). Jutzi et al. (2019) used a mouse model to show that mutations in *Nfe2* promotes the development of myelosarcoma and/or AML. Furthermore, *Nfe2* may act by enhancing the expression of hematopoietic master regulators, such as SCL/TAL1 and GATA2 (Siegwart et al., 2020), both of which were found to be important module genes in the current study. Furthermore, we showed *NFE2* to be highly expressed across all AML subtypes and its expression significantly correlated with methylation levels in AML patients. *Trim27* encodes a member of the tripartite motif (TRIM) family and is localized in the nuclear matrix. In addition, it represses gene transcription by interacting with the enhancer of polycomb protein. A recent article has shown that USP7 and TRIM27 are integral components of PRC1.1 and USP7-TRIM27 axis is a druggable target in leukemia (Maat et al., 2021). A study by Ma et al. (2019) revealed that overexpression of *TRIM27* suppresses apoptosis of esophagus cancer cells and increased their glucose uptake. Transcriptome analysis of *Trim27*-overexpressing myeloid progenitor cells indicated that it increased the expression of myelopoiesis regulators, myeloid proliferation-related signaling genes, and myeloid maturation-related genes (Wang et al., 2018). MEF2C, a TF, is encoded by the member of the MADS box transcription enhancer factor 2 (*MEF2*) family of proteins and is known to play a role in myogenesis. High expression of MEF2C has been shown to be associated with adverse-risk features and poor outcome in pediatric and adult AML (Cante-Barrett et al., 2014; Laszlo et al., 2015; Xu et al., 2020c). In the current study, we also observed the upregulation of *Mef2c* in both mouse and human AML compared to healthy control. Aberrant phosphorylation of MEF2C has been reported to induce chemotherapy resistance in AML (Brown et al., 2018). *Ets1* encodes a member of the ETS family of transcription factors, which are defined by the presence of a conserved ETS DNA-binding domain. In hematopoietic cells, *Ets1* regulates cellular differentiation, and in other cells, such as endothelial, vascular smooth muscle and epithelial cancer cells, it promotes invasive behavior (Dittmer, 2003). Our study showed that *Ets1* is downregulated in both human and mouse AML and harbors a frame-shift mutation in D2 mice. The ETS family of proteins function either as transcriptional activators or repressors. A study by Fu et al. (2019) has demonstrated that *Ets1* plays a crucial role in MLL/EB1-mediated leukemic transformation in a mouse bone marrow transplantation model. Moreover, the expression of *ETS1* was found to be significantly correlated with its methylation levels in AML patients in the current study. Our study has a few limitations. While we identified important hub genes in leukemia based on various analyses and established their functional and clinicopathological importance in AML, their expression and the underlying mechanisms need to be experimentally verified.

We used a cross-species integrative approach to identify the key hub genes in AML. The co-expression network constructed using human expression data and differential analysis using mouse expression data identified a total of 412 genes that were found to be involved in functions and pathways related to hematopoiesis, immune-system physiology, and leukemia. The integration of protein interaction information, gene functions, regulatory, and mutation data identified multiple key hub genes, particularly *Nfe2*, *Trim27*, *Mef2c*, *Ets1*, *Tal1*, *Foxo1*, and *Gata1* in AML. These genes can be further explored to understand their detailed mechanisms of function in AML.

## Data Availability

Publicly available datasets were analyzed in this study. This data can be found here: The dataset analyzed for this study can be found in the GeneNetwork with the accession GN144 (http://gn1.genenetwork.org/webqtl/main.py?FormID=sharinginfo&GN_AccessionId=144), and NCBI-GEO database (https://www.ncbi.nlm.nih.gov/geo/query/acc.cgi?acc=GSE9476; https://www.ncbi.nlm.nih.gov/geo/query/acc.cgi?acc=GSE13690; https://www.ncbi.nlm.nih.gov/geo/query/acc.cgi?acc=GSE14924).
