# Supplementary material for: Weighted gene co-expression network analysis identifies key hub genes and pathways in acute myeloid leukemia
Source: Front Genet. 2023 Feb 27;14:1009462. doi: 10.3389/fgene.2023.1009462 (PMC10008864; doi:10.3389/fgene.2023.1009462)
Supplement: Supplementary file 4 [file Image1.pdf]

# *Supplementary Material*

## Supplementary Figures

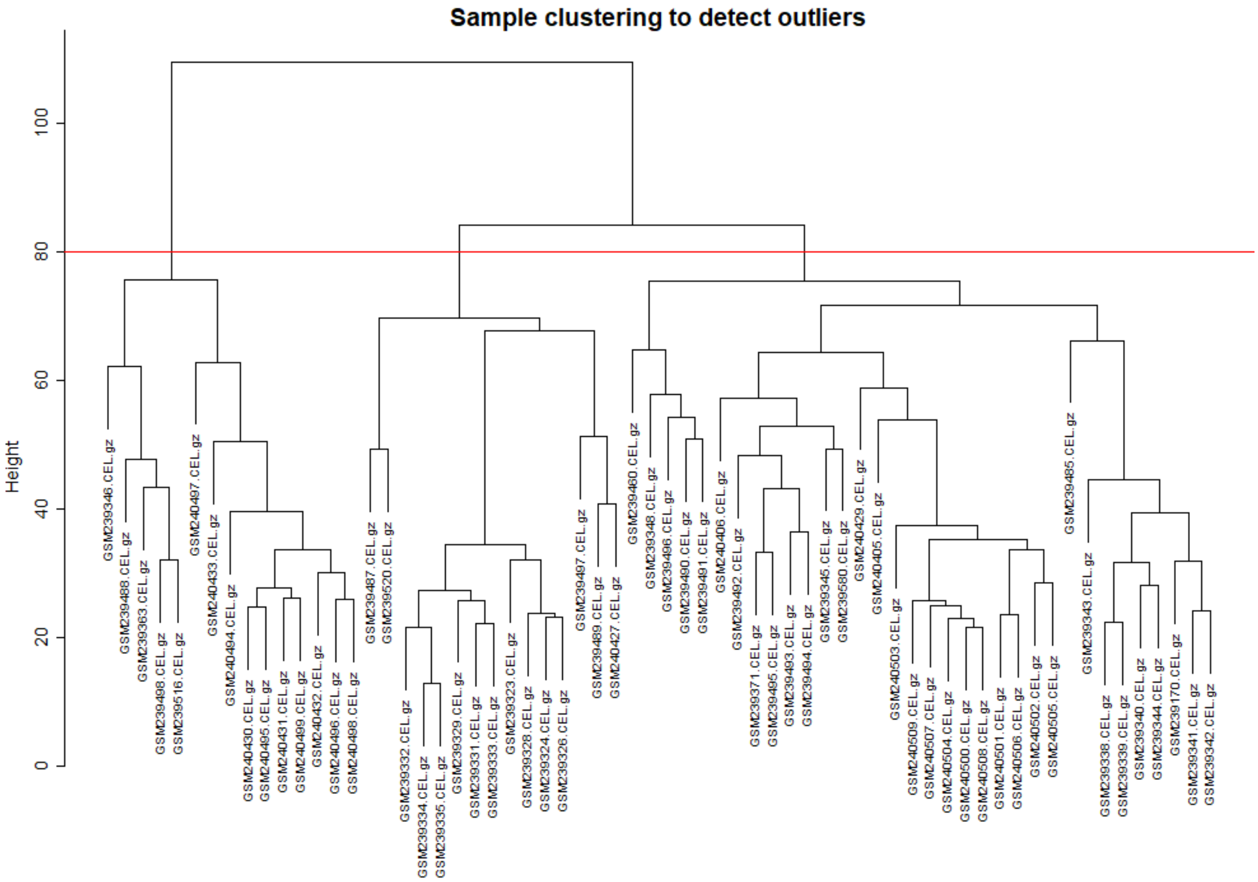


**Supplementary Figure 1. Detection of outliers in samples (GSE9476) considered for WGCNA analysis.** The red line indicates height where the tree was cut. Cutting the tree resulted in 3 clusters, two containing 15 samples each and one containing 34 samples. None of the samples were categorized as 'cluster 0', indicating no extreme outliers.

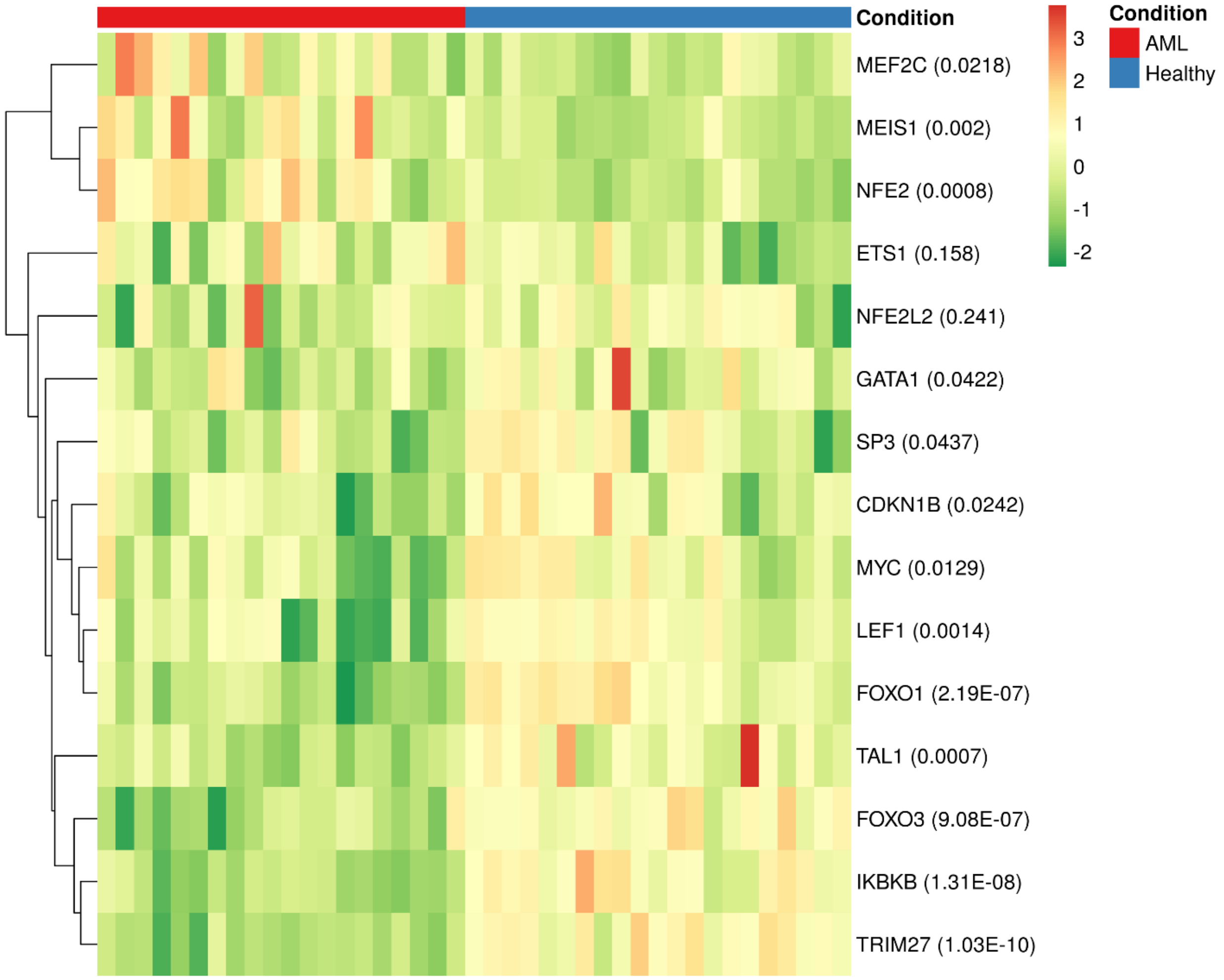


**Supplementary Figure 2. Validation of the differential expression of important candidate genes between AML and control samples using an independent human microarray study (GSE14924).** The values inside the parenthesis next to gene name indicate B&H adjusted p-value. All the genes except NFE2L2 and ETS1 were significantly differentially expressed between human AML and healthy samples.

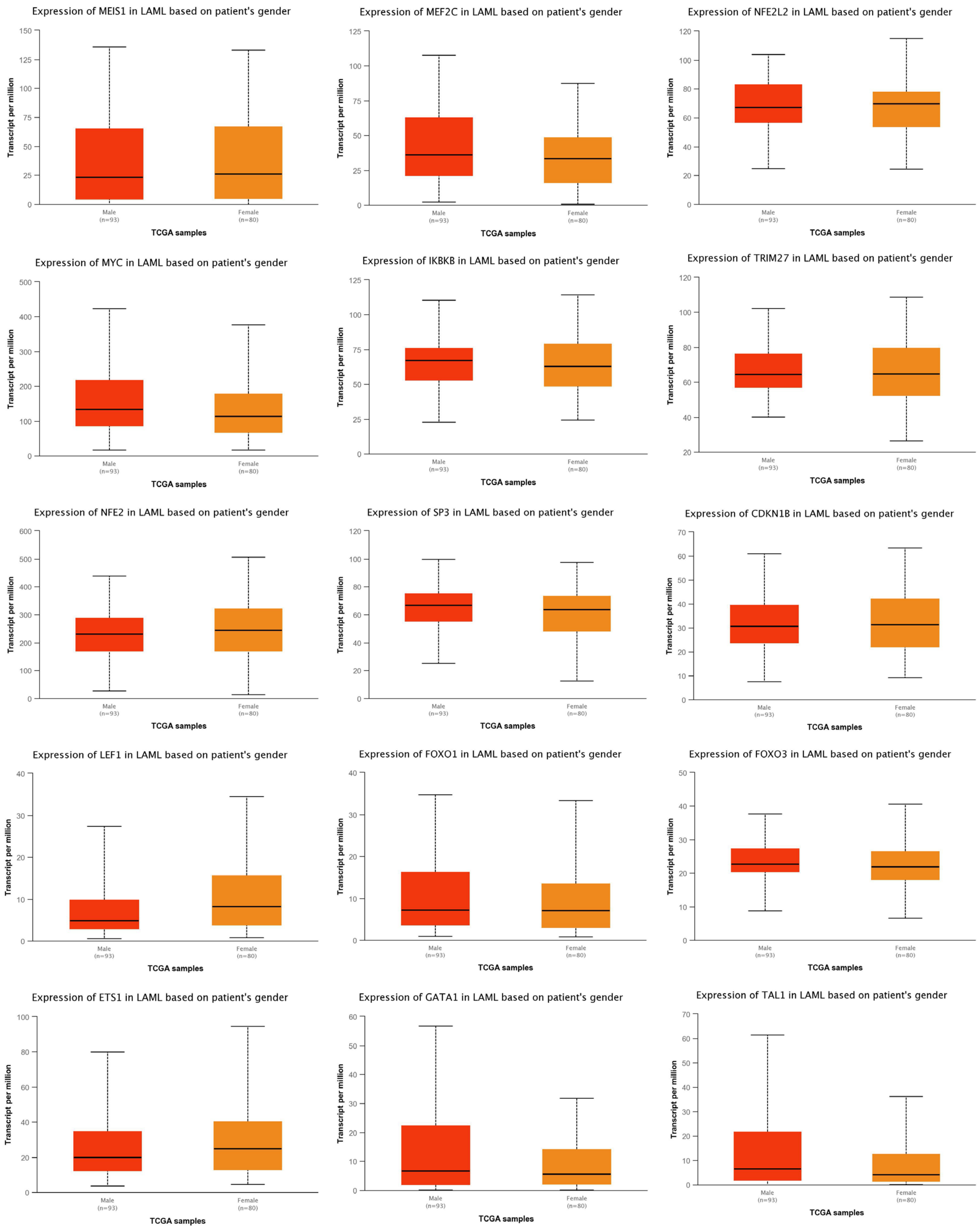


**Supplementary Figure 3. Expression of the important candidate genes based on gender of AML patients.** The analysis was based on TCGA expression data. Left box-plot indicates male, whereas the right plot indicates the female values. The difference in expression between the gender was only significant for TAL1 gene ($P$ = 0.0275). LAML: Acute Myeloid Leukemia.

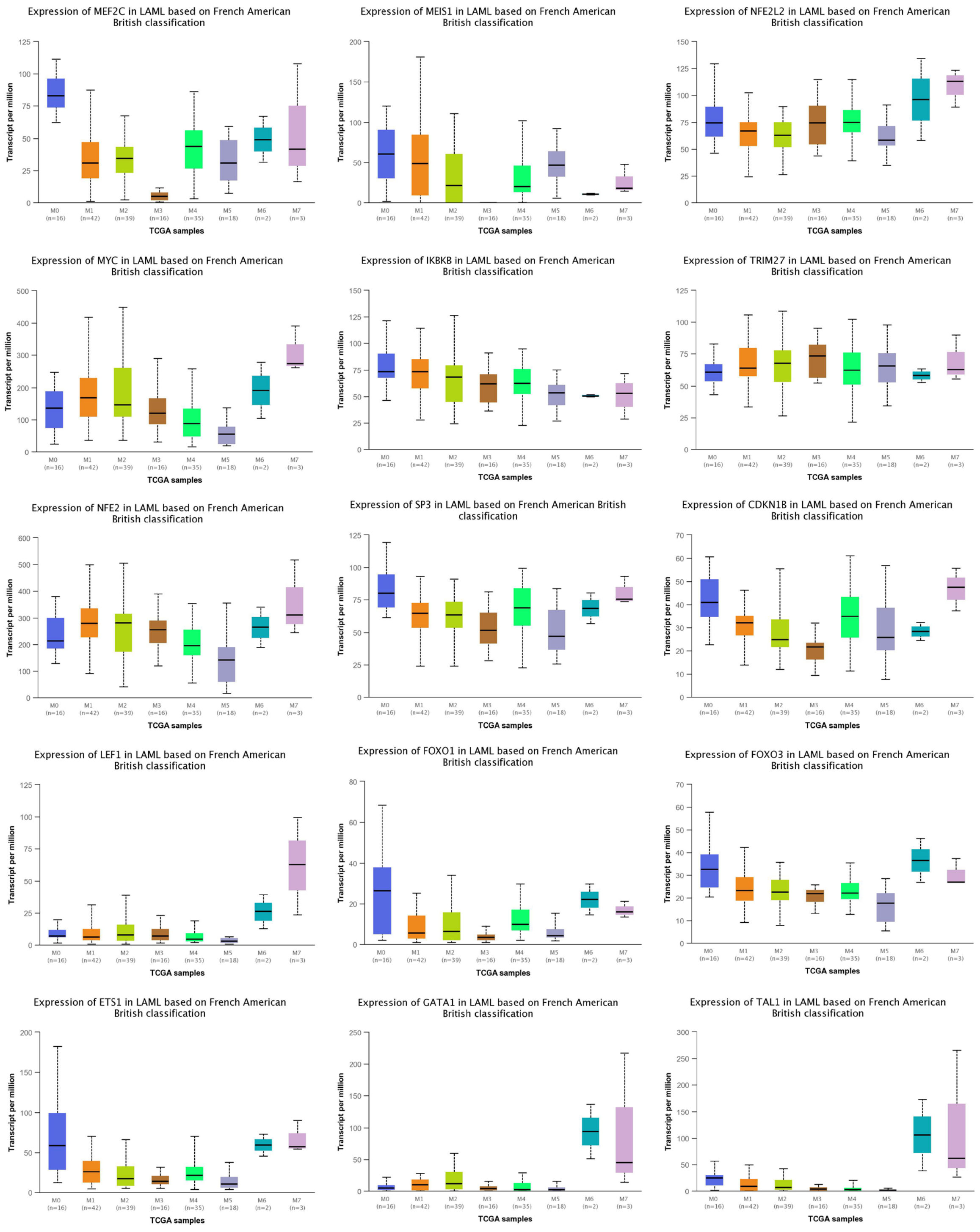


**Supplementary Figure 4. Expression of the important candidate genes across different subtypes/stages of AML.** The analysis was based on TCGA expression data. LAML: Acute Myeloid Leukemia. Subtypes M0 through M5 all start in immature forms of white blood cells. M6 AML starts in very immature forms of red blood cells, while M7 AML starts in immature forms of cells that make platelets (https://www.cancer.org/cancer/acute-myeloid-leukemia/detection-diagnosis-staging/how-classified.html).

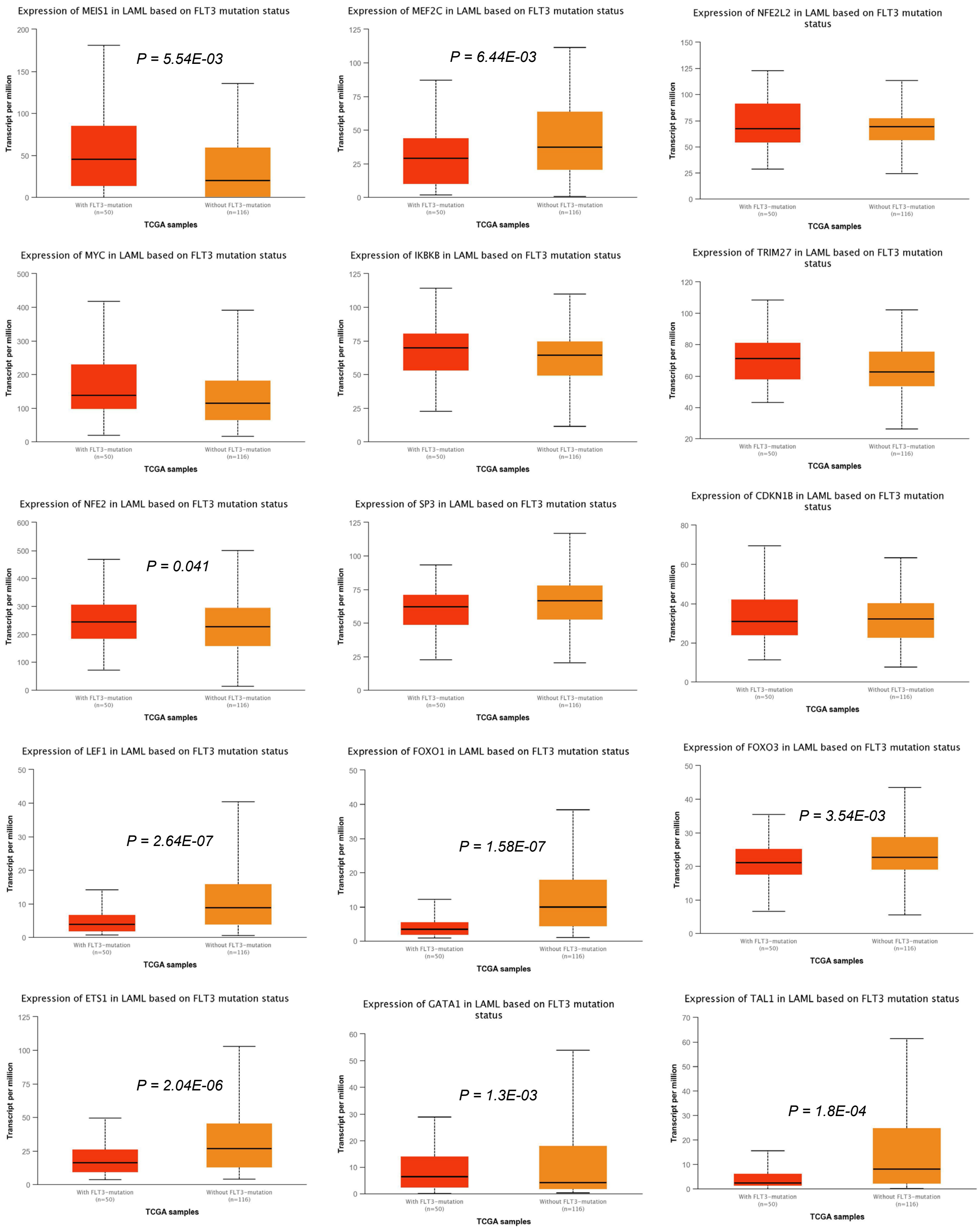


**Supplementary Figure 5: Expression of the important candidate genes based on FLT3 mutation status of AML patients.** The analysis was based on TCGA expression data. Significant differences are indicated with a *P-value*. Left box-plot indicates samples with FLT3 mutation, whereas the right plot indicates samples without mutation. LAML: Acute Myeloid Leukemia.

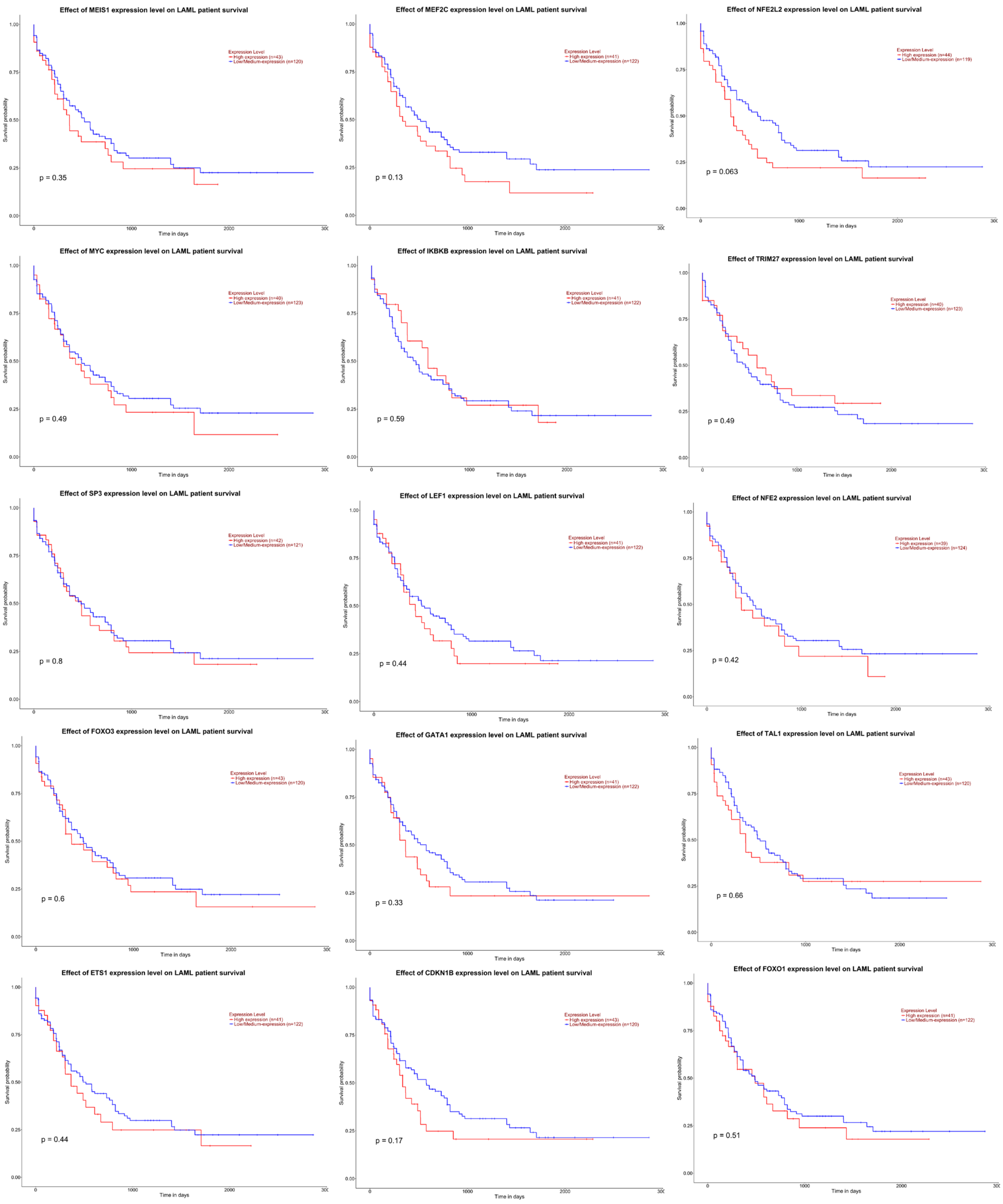


**Supplementary Figure 6: Overall survival analysis of the AML patients based on expression of the important candidate genes.** The analysis was based on TCGA expression and survival data of AML patients. LAML: Acute Myeloid Leukemia.

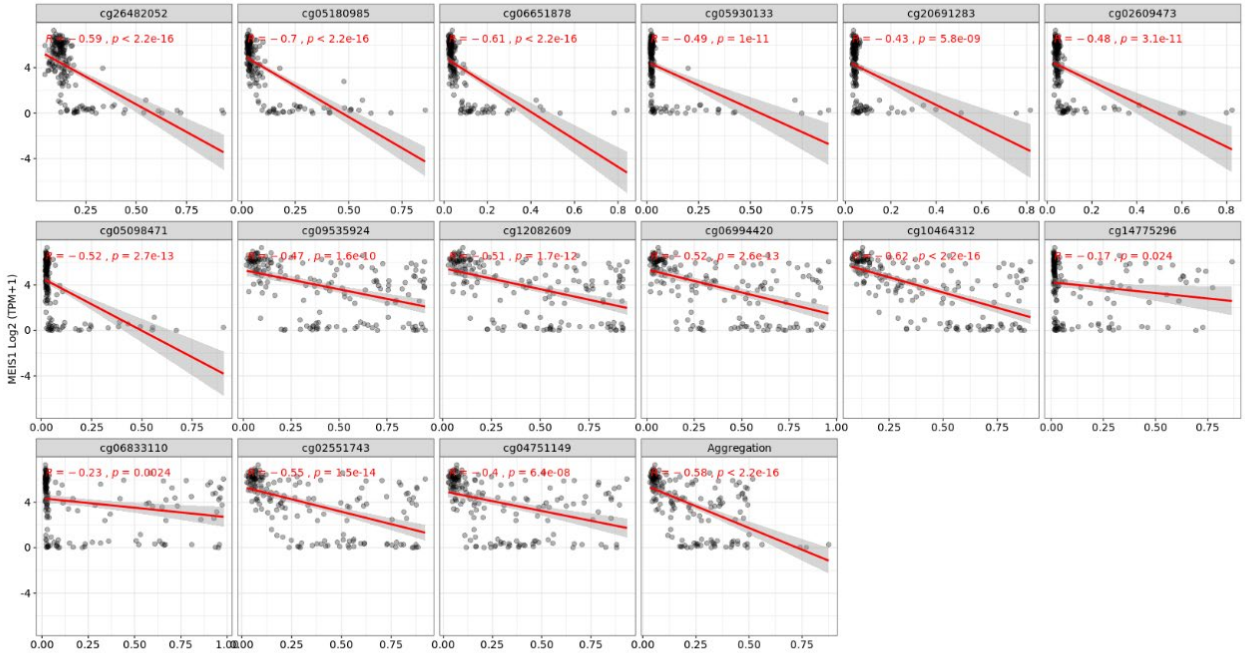


**Supplementary Figure 7: Correlation between MEIS1 expression and methylation.** Correlation between the gene expression and methylation Beta-value of individual probe is shown in each plot. "Aggregation" plot represents the mean methylation for all the individual CpGs that were selected. Probes specific to 'island' CpGs were selected (all probes were selected when 'island' specific probes were not there). Source

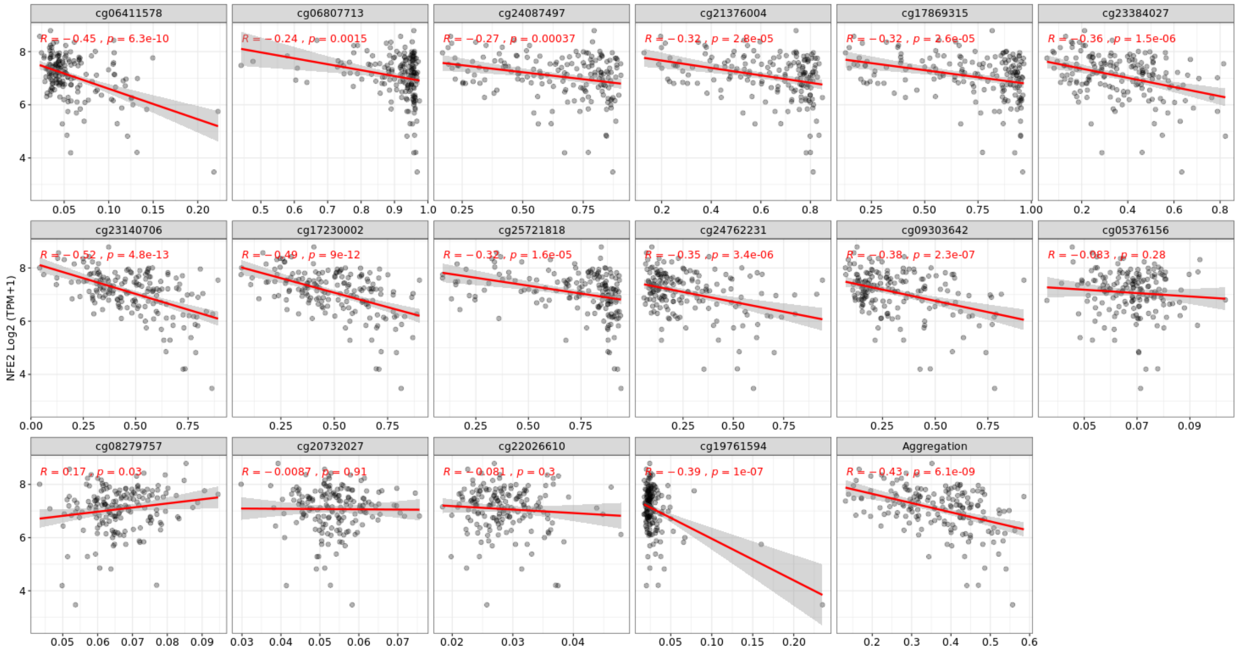


**Supplementary Figure 8: Correlation between NFE2 expression and methylation.** Correlation between the gene expression and methylation Beta-value of individual probe is shown in each plot. "Aggregation" plot represents the mean methylation for all the individual CpGs that were selected. Probes specific to 'island' CpGs were selected (all probes were selected when 'island' specific probes were not there).

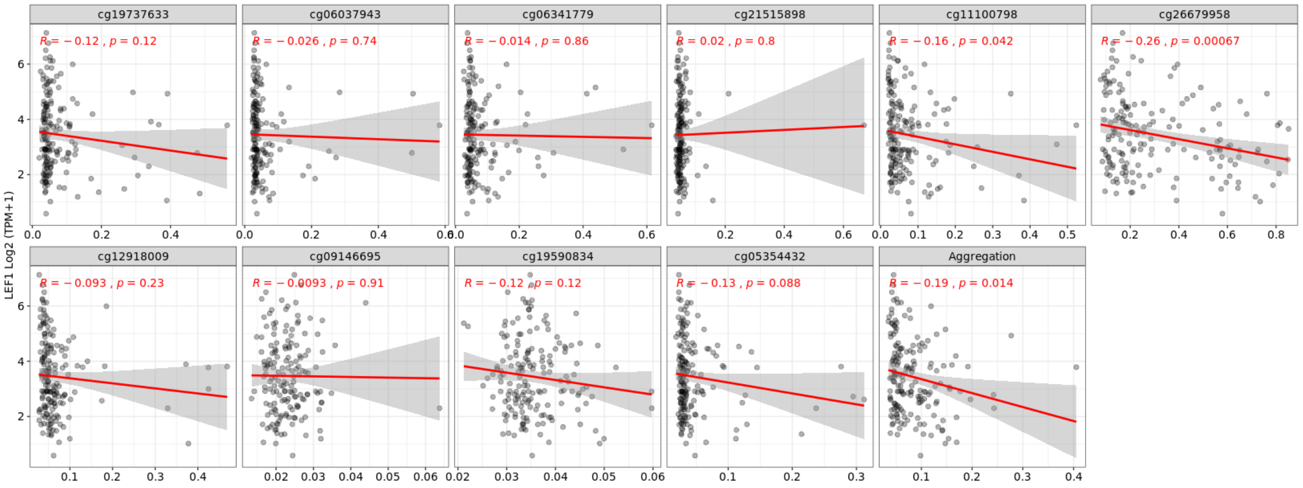


**Supplementary Figure 9: Correlation between LEF1 expression and methylation.** Correlation between the gene expression and methylation Beta-value of individual probe is shown in each plot. "Aggregation" plot represents the mean methylation for all the individual CpGs that were selected. Probes specific to 'island' CpGs were selected (all probes were selected when 'island' specific probes were not there).

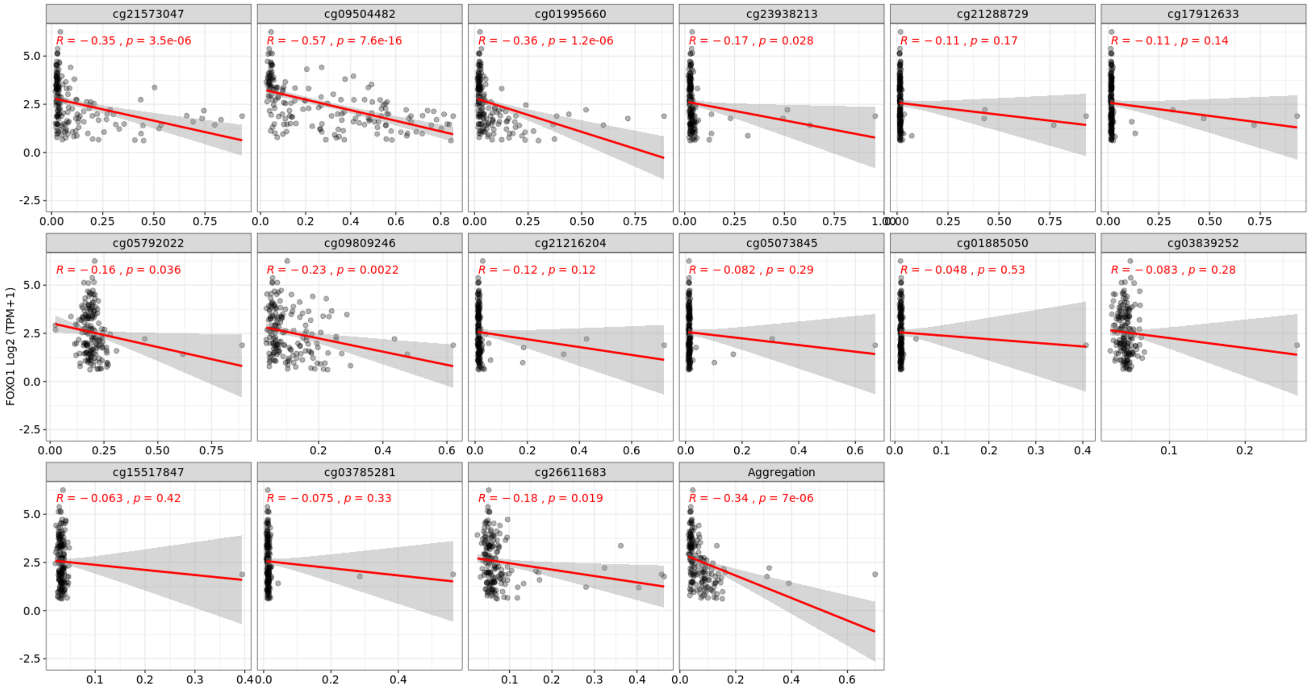


**Supplementary Figure 10: Correlation between FOXO1 expression and methylation.** Correlation between the gene expression and methylation Beta-value of individual probe is shown in each plot. "Aggregation" plot represents the mean methylation for all the individual CpGs that were selected. Probes specific to 'island' CpGs were selected (all probes were selected when 'island' specific probes were not there).

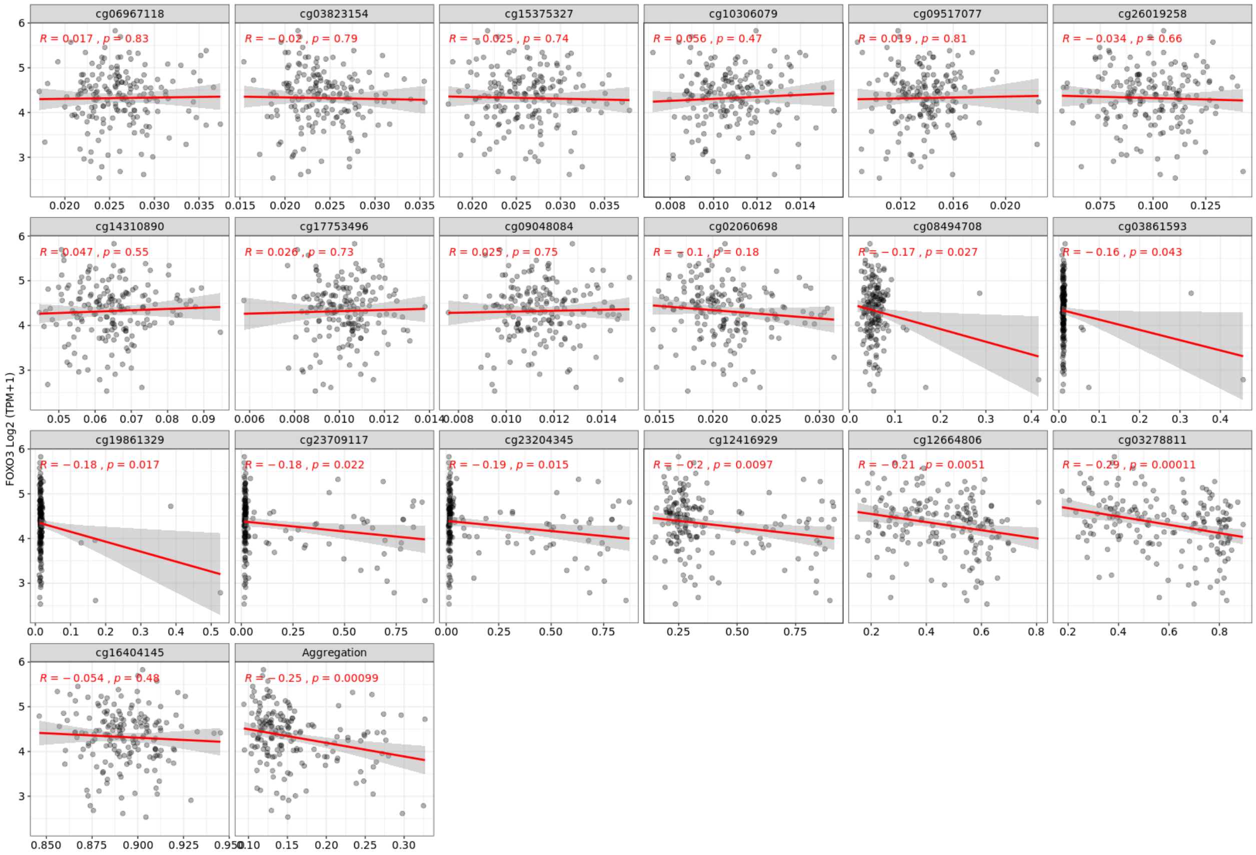


**Supplementary Figure 11: Correlation between FOXO3 expression and methylation.** Correlation between the gene expression and methylation Beta-value of individual probe is shown in each plot. "Aggregation" plot represents the mean methylation for all the individual CpGs that were selected. Probes specific to 'island' CpGs were selected (all probes were selected when 'island' specific probes were not there).

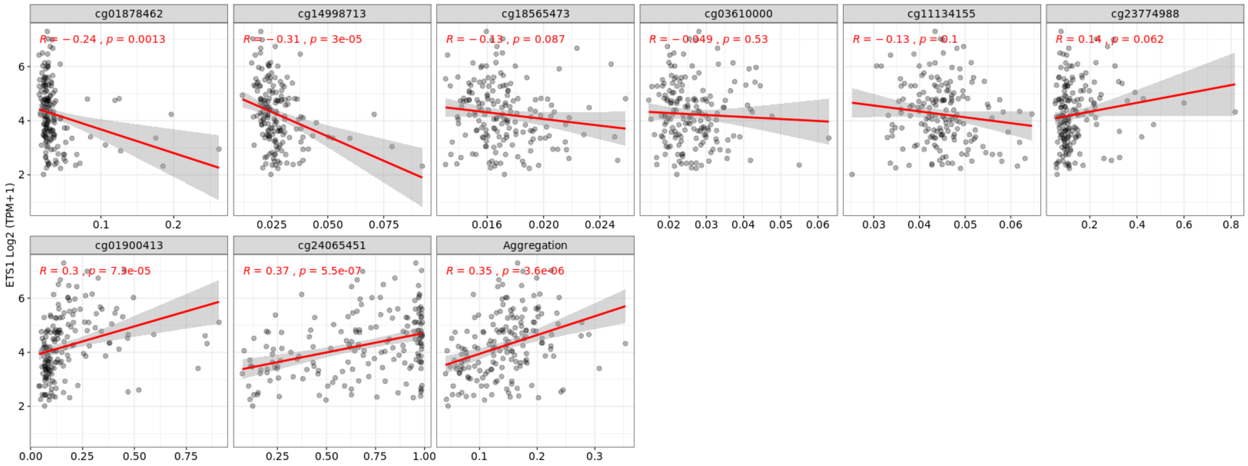


**Supplementary Figure 12: Correlation between ETS1 expression and methylation.** Correlation between the gene expression and methylation Beta-value of individual probe is shown in each plot. "Aggregation" plot represents the mean methylation for all the individual CpGs that were selected. Probes specific to 'island' CpGs were selected (all probes were selected when 'island' specific probes were not there).

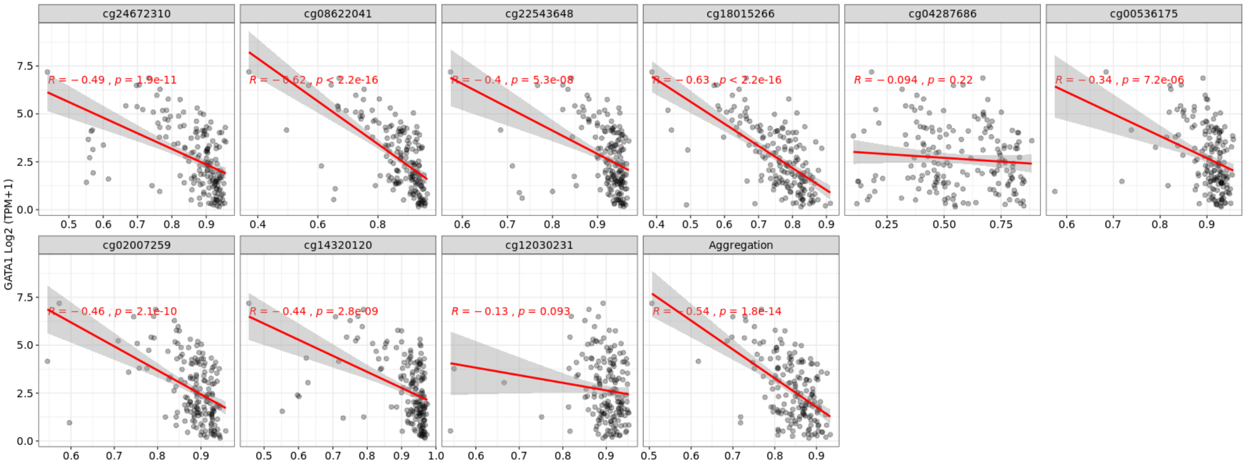


**Supplementary Figure 13: Correlation between GATA1 expression and methylation.** Correlation between the gene expression and methylation Beta-value of individual probe is shown in each plot. "Aggregation" plot represents the mean methylation for all the individual CpGs that were selected. Probes specific to ‘island’ CpGs were selected (all probes were selected when ‘island’ specific probes were not there).
